# Spatially correlated classical and quantum noise in driven qubits

**DOI:** 10.1038/s41534-024-00842-9

**Published:** 2024-04-30

**Authors:** Ji Zou, Stefano Bosco, Daniel Loss

**Affiliations:** Department of Physics, University of Basel, Basel, Switzerland

**Keywords:** Quantum dots, Qubits

## Abstract

Correlated noise across multiple qubits poses a significant challenge for achieving scalable and fault-tolerant quantum processors. Despite recent experimental efforts to quantify this noise in various qubit architectures, a comprehensive understanding of its role in qubit dynamics remains elusive. Here, we present an analytical study of the dynamics of driven qubits under spatially correlated noise, including both Markovian and non-Markovian noise. Surprisingly, we find that by operating the qubit system at low temperatures, where correlated quantum noise plays an important role, significant long-lived entanglement between qubits can be generated. Importantly, this generation process can be controlled on-demand by turning the qubit driving on and off. On the other hand, we demonstrate that by operating the system at a higher temperature, the crosstalk between qubits induced by the correlated noise is unexpectedly suppressed. We finally reveal the impact of spatio-temporally correlated 1/*f* noise on the decoherence rate, and how its temporal correlations restore lost entanglement. Our findings provide critical insights into not only suppressing crosstalk between qubits caused by correlated noise but also in effectively leveraging such noise as a beneficial resource for controlled entanglement generation.

## Introduction

Quantum computers hold great promise for solving computational problems that are intractable for classical ones, due to their ability to exploit the quantum coherence of qubits^1,2^. However, quantum coherence is extremely fragile and noise poses a major challenge to quantum information processing^3,4^. A comprehensive understanding of the effects of noise is the first step towards the development of effective noise mitigation strategies, and therefore, is crucial to leverage the full potential of large-scale quantum processors^5,6^.

In the single-qubit scenario, the noise experienced by the qubit is local and can be characterized by a single noise spectrum^7,8^. It has been experimentally measured in different quantum computation architectures, including spin qubits in semiconductors^9,10^, nitrogen-vacancies^11,12^, trapped ions^13,14^, and superconducting quantum circuits^15–18^, by employing different noise spectroscopy protocols. While the effect of single-qubit noise, in particular 1/*f* noise in solid state architectures, is still being actively investigated in order to develop better strategies for mitigating its impact^19–23^, we have gained a relatively good understanding of its characteristics^24–30^. Various techniques to address it in quantum computation have been proposed, including decoherence-free sweet spots^31–36^, quantum error correction codes^37–40^, dynamical decoupling^41–44^, and optimal control methods^45–51^, which have shown promise in reducing the impact of single-qubit noise and improving the performance of quantum devices.

However, the presence of spatially correlated noise can limit the applicability of proposed protocols in multi-qubit settings. For instance, major quantum error-correcting codes rely on independently detecting and correcting errors on individual qubits^37–40^. Correlated noise across multiple qubits impedes the effectiveness of these codes, leading to a higher probability of errors remaining undetected and reducing the performance of quantum systems^52–56^. It is therefore crucial to better quantify and understand correlated noise. This has stimulated various theoretical proposals^57–60^ and experimental works in the measurement of correlated noise, for example, in architectures based on spin qubits^61,62^ and superconducting qubits^63^. However, despite the progress in quantifying spatial noise correlations, a comprehensive understanding of how they affect the performance of multiqubit systems is still lacking.

On the other hand, while spatially correlated noise can have detrimental effects on quantum systems, it raises the question of whether the correlations stored in the noise can be harnessed to process quantum information^64,65^. For instance, correlated noise offers the intriguing possibility to imprint the correlations onto a two-qubit system, effectively converting the correlation of noise into entanglement between the qubits. To develop effective strategies either for mitigating correlated noise or for leveraging the correlations it stores, a comprehensive understanding of the effects of correlated noise in multiqubit settings is required.

In this work, we present a systematic theoretical investigation of the impact of spatially correlated noise on the dynamics of driven qubits with a focus on their entanglement, considering both temporally correlated and uncorrelated noise. We provide analytical solutions for all discussed scenarios, accompanied by detailed derivations in the Supplementary Information to guarantee the high reproducibility of all our results. We uncover several interesting findings as summarized in Table 1. In particular, we find that operating qubits at a temperature higher than the Rabi frequency effectively mitigates the detrimental effects of correlated noise, notably reducing crosstalk between qubits. This unexpected decrease in crosstalk at elevated temperatures aligns with recent experimental findings in spin qubits^66^, highlighting a practical approach to managing the harmful effects of correlated noise. Besides, we show that, to leverage the beneficial aspect of correlated noise, such as generating significant long-lived entanglement, it is essential to drive the qubits at lower temperatures. A critical aspect of this finding is the ability to control this entanglement generation on-demand by activating or deactivating the qubit driving.

**Table 1:** Summary of Key Findings

Qubit drive	Noise	Low T (Quantum noise plays a role)	High T (Classical noise dominates)
Without drive	Pure-dephasing noise	Correlated dephasing process does not generate long-lived entanglement	Qubits do not entangle; Correlated noise modifies *dephasing rate*
With drive	Transverse noise	Correlated relaxation process does generate *significant long-lived entanglement*	Qubits do not entangle; Correlated noise modifies *relaxation rate*

The section of Results is organized as follows. We first introduce the model Hamiltonian. Next, we discuss the concept of local and spatially correlated noise spectral densities, distinguishing their classical and quantum components. We then extend the commonly used master equation for single qubit dynamics to a set of more broadly applicable master equations. These are tailored for the driven dynamics of two-qubit systems in the presence of spatially correlated generic noise, which sets the stage for the further discussion.

We then investigate spatially correlated 1/*f* noise in pure dephasing dynamics without coherent drives. We pay particular attention to how the classical and quantum components participate in the two-qubit dynamics and whether they can be exploited to generate entanglement. We demonstrate that the classical correlations in the noise affect the dynamics through correlated pure dephasing, which modifies the dephasing rate but does not induce any coherence. In contrast, our findings show that correlated quantum noise influences two-qubit dynamics via noise-induced coherent interactions between qubits and correlated pure-dephasing processes. Notably, while the former facilitates converting noise correlation into entanglement, the latter does not.

Next, we present an analytical study of the two-qubit dynamics under the influence of spatially-correlated Markovian transverse noise with coherent drives. Our analysis reveals that the quantum noise correlations induce a coherent symmetry exchange interaction, a Dzyaloshinskii-Moriya interaction between the two qubits, and a correlated decoherence process. We observe an intriguing interplay between these ingredients, resulting in distinct dynamical phases that can be achieved with different parameter values. Contrary to pure dephasing, we discover that both coherent interactions and correlated decoherence not only generate substantial entanglement but also ensure its longevity. This long-lived entanglement, enhanced by the correlated nature of the process, shows promise for implementing two-qubit gates and advancing quantum information processing tasks.

Finally, we conduct an analytical investigation into the influence of correlated 1/*f* noise on the dynamics of driven qubits. Our study shows that the non-Markovianity of the 1/*f* noise results in effective time-dependent decoherence rate that exhibits temporary negative values for some time intervals^67^. We focus on the classical and quantum correlated 1/*f* noise. We find that the classical spatially-correlated 1/*f* noise is still unable to generate any entanglement. However, the non-trivial temporal correlations of the noise can restore the coherence lost in the environment. On the other hand, the non-Markovian nature of the quantum correlated 1/*f* noise leads to a temporary decrease of the entanglement generated by the quantum noise. We also compare our findings with the single qubit scenario, highlighting the different effects of correlated noise on idle qubits and on qubits with coherent drives.

## Results

### Hamiltonian

**Fig. 1:**
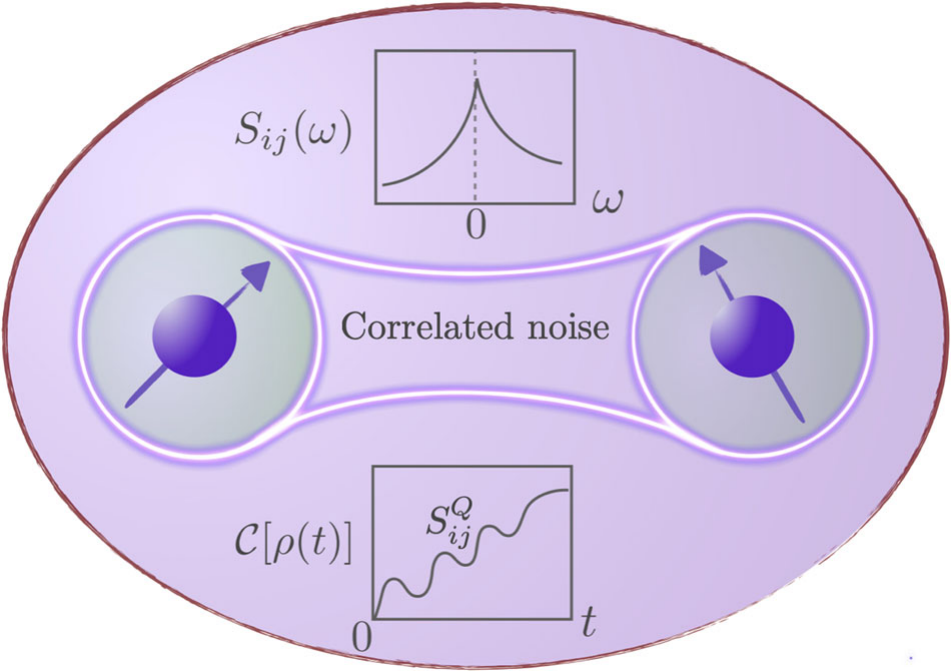
A schematic for two qubits in an environment experiencing both local and spatially correlated noise. The noise is quantified by the cross-noise power spectral densities *S*_*ij*_(*ω*), with its positive and negative frequency parts measuring the ability of qubits to emit and absorb energy, respectively. The asymmetric nature of the noise spectral density, in the presence of quantum noise, is linked to the asymmetry between the absorption and emission processes. The quantum correlated noise can be harnessed to generate entanglement.

In this work, we analyse the dynamics of two qubits that are driven and are situated in the same environment, as depicted in Fig. 1. The qubits are subjected to both local and spatially-correlated (non-local) noise, which can have either a classical or quantum nature and can be either temporally correlated (such as 1/*f*) or uncorrelated ("Markovian”). We describe the combined system with the following Hamiltonian:

1 $$H(t)={H}_{{{{\rm{S}}}}}+{H}_{{{{\rm{drive}}}}}(t)+{H}_{{{{\rm{SE}}}}}+{H}_{{{{\rm{E}}}}},$$

where *H*_S_ is the Hamiltonian of the two qubits that are characterized by qubit-frequency splittings Δ_*i*_ (*i* = 1, 2), and *H*_drive_(*t*) describes the time-dependent driving of the two qubits, *H*_E_ is the Hamiltonian of the environment, and *H*_SE_ describes the coupling between the two qubits and the environment. We assume the two qubits are driven coherently at frequency *ω*_*di*_ with the drive amplitude *ℏ*Ω_*i*_. We then consider the following Hamiltonian:

2 $${H}_{{{{\rm{S}}}}}+{H}_{{{{\rm{drive}}}}}(t)=\mathop{\sum}\limits_{i=1,2}\left[\frac{\hslash {\Delta }_{i}}{2}{\sigma }_{i}^{z}+\hslash {\Omega }_{i}\cos ({\omega }_{di}t){\sigma }_{i}^{x}\right],$$

where ${\sigma }_{i}^{x,z}$ are the Pauli matrices for qubit *i* and *ℏ* is the Planck’s constant. We describe the environment with

3 $${H}_{{{{\rm{E}}}}}=\mathop{\sum}\limits_{{{{\bf{k}}}}}\hslash {\omega }_{{{{\bf{k}}}}}{b}_{{{{\bf{k}}}}}^{{\dagger} }{b}_{{{{\bf{k}}}}},$$

where *b*_**k**_ and ${b}_{{{{\bf{k}}}}}^{{\dagger} }$ are operators describing quasiparticles in the environment leading to the decoherence of the qubits, such as phonons in semiconductors^68–70^ or magnons in hybrid systems^71–80^. While we leave the spectrum *ω*_**k**_ unspecified which can be either linear, for example, for acoustic phonons or quadratic for magnons, we specialize to single axis qubit-environment couplings:

4 $${H}_{{{{\rm{SE}}}}}=\mathop{\sum}\limits_{i=1,2}{\sigma }_{i}^{z}{E}_{i},\quad {{{\rm{with}}}}\quad {E}_{i}\equiv {g}_{{{{\bf{k}}}}}{e}^{i{{{\bf{k}}}}\cdot {{{{\bf{r}}}}}_{i}}{b}_{{{{\bf{k}}}}}+\,{{\mbox{H.c.}}}\,,$$

implying that pure-dephasing dynamics dominates the decoherence process in the absence of coherent drives. Here, *E*_*i*_ are operators acting on the environment Hilbert space, **r**_*i*_ is the positions of the *i*th qubit, and *g*_**k**_ is the coupling strength.

When the system is subjected to coherent drives, we effectively rotate the quantization axis in a frame rotating at the driving frequencies, where the qubits can exchange not only information with the environment but also exchange energy by emitting and absorbing quasiparticles. To illustrate this, we perform the unitary transformation $R(t)=\exp (i{\omega }_{d1}{\sigma }_{1}^{z}t/2)\otimes \exp (i{\omega }_{d2}{\sigma }_{2}^{z}t/2)$. The Hamiltonian in the rotating frame then is given by ${{\mathcal{H}}}=i\hslash {\partial }_{t}R{R}^{{\dagger} }+RH{R}^{{\dagger} }$. We note that the unitary operator *R*(*t*) commutes with *H*_SE_ and *H*_E_, leaving them invariant in the rotating frame. After applying the rotating-wave approximation by neglecting counter-rotating terms that oscillate fast at frequency 2*ω*_*di*_, the total Hamiltonian reads,

5 $${{{\mathcal{H}}}}={{{{\mathcal{H}}}}}_{{{{\rm{S}}}}}+{H}_{{{{\rm{SE}}}}}+{H}_{{{{\rm{E}}}}},$$

where the qubit Hamiltonian in the new rotating frame is ${{{{\mathcal{H}}}}}_{{{{\rm{S}}}}}={\sum }_{i = 1,2}\hslash \left({\delta }_{i}{\sigma }_{i}^{z}+{\Omega }_{i}{\sigma }_{i}^{x}\right)/2$ with the detuning *δ*_*i*_ = Δ_*i*_ − *ω*_*di*_. When the qubits are driven at resonance *ω*_*di*_ = Δ_*i*_, we arrive at:

6 $${{{{\mathcal{H}}}}}_{{{{\rm{S}}}}}=\frac{\hslash \Omega }{2}\mathop{\sum}\limits_{i}{\hat{\sigma}}_{i}^{z},\quad {{{\rm{and}}}}\quad {H}_{{{{\rm{SE}}}}}=-\mathop{\sum}\limits_{i}{\hat{\sigma }}_{i}^{x}{E}_{i}.$$

Here, we have rotated the axis in the spin space, ${{{{\mathcal{H}}}}}_{{{{\rm{S}}}}}\to {R}_{y}{{{{\mathcal{H}}}}}_{{{{\rm{S}}}}}{R}_{y}^{{\dagger} }$ with ${R}_{y}=\exp [i(\pi /2){\sigma }_{y}/2]$, such that the qubit quantization axis is aligned with the *z* axis, and we label this new basis with Pauli matrices ${\hat{\sigma }}_{i}$. We also assume here that the driving strength is equal for both qubits, denoting it as Ω ≡ Ω_1_ = Ω_2_. We remark that, in this scenario, the relaxation dynamics dominates the decoherence process, which has been exploited for noise-spectroscopy applications to extract noise spectra near frequency Ω^63^. We focus our analysis in this work on the effects of correlated noise in two fundamental scenarios: one in the absence of coherent drives where pure-dephasing noise dominates, and the other in the presence of resonant drive where the transverse noise is prominent. We point out that while we have concretely assumed an environment consisting of quasiparticles described by operators ${b}_{k},{b}_{k}^{{\dagger} }$, the dissipative effects depend solely on the noise spectral density of the environment. Motivated by experimental works^35,81,82^, we choose this to be 1/f in our later discussions, which enables us to accurately capture the dynamics of the qubits, even though the microscopic origin of the noise may vary. For example, it could be due to an ensemble of correlated two-level fluctuators.

### Distinguishing classical from quantum noise in local and spatially correlated noise

We described the qubit Hamiltonian under external coherent drives in the preceding section. Here, we discuss the noise experienced by the qubits due to their coupling to the environment, focusing on both local and spatially correlated noise while distinguishing their classical and quantum natures. We define the usual two-point noise correlation function in time domain^8^:

7 $${S}_{ij}(t)\equiv \langle {E}_{i}(t){E}_{j}(0)\rangle ,$$

where $E(t)\equiv {e}^{i{H}_{{{{\rm{E}}}}}t/\hslash }E{e}^{-i{H}_{{{{\rm{E}}}}}t/\hslash }$ and $\langle {{{\mathcal{O}}}}\rangle \equiv {{{\rm{tr}}}}({\rho }_{{{{\rm{B}}}}}{{{\mathcal{O}}}})$ with the thermal state ${\rho }_{{{{\rm{B}}}}}={e}^{-\beta {H}_{{{{\rm{E}}}}}}/{{{\rm{tr}}}}[\exp (-\beta {H}_{{{{\rm{E}}}}})]$ and *β* = 1/*k*_B_*T*, where *T* is the temperature and *k*_B_ is the Boltzmann constant. The noise power spectral density is given by the Fourier transformation of the correlation function:

8 $${S}_{ij}(\omega )=\int\nolimits_{-\infty }^{\infty }dt\,{e}^{i\omega t}{S}_{ij}(t).$$

Here, *S*_*ii*_(*ω*) with *i* = {1, 2} is the auto power spectral density standing for the local noise, whereas *S*_*ij*_(*ω*) with *i* ≠ *j* is the cross power spectral density representing spatially correlated noise. We note that ${S}_{ij}(\omega )={S}_{ji}^{* }(\omega )$, indicating that the local noise spectral density is a real-valued function while the correlated noise can be complex-valued. This is also clear from the explicit expression of the noise spectral density:

9 $$\begin{array}{l}{S}_{ij}(\omega )\,=\,2\pi \mathop{\sum}\limits_{{{{\bf{k}}}}}| {g}_{{{{\bf{k}}}}}{| }^{2}{e}^{-i{{{\bf{k}}}}\cdot ({{{{\bf{r}}}}}_{j}-{{{{\bf{r}}}}}_{i})}\left[{n}_{{{{\rm{B}}}}}({\omega }_{{{{\bf{k}}}}})+1\right]\delta (\omega -{\omega }_{{{{\bf{k}}}}})\\\qquad\quad+\,2\pi \mathop{\sum}\limits_{{{{\bf{k}}}}}| {g}_{{{{\bf{k}}}}}{| }^{2}{e}^{i{{{\bf{k}}}}\cdot ({{{{\bf{r}}}}}_{j}-{{{{\bf{r}}}}}_{i})}{n}_{{{{\rm{B}}}}}({\omega }_{{{{\bf{k}}}}})\delta (\omega +{\omega }_{{{{\bf{k}}}}}),\end{array}$$

where *n*_B_(*ω*) = 1/(*e*^*βℏω*^ − 1) is the Bose-Einstein distribution and the spatial vector **r**_*j*_ − **r**_*i*_ connects the positions of qubit *j* and *i*. The cross power spectral density *S*_12_(*ω*) is real when the spectrum of the environment is symmetric in momentum *ω*_**k**_ = *ω*_−**k**_, whereas it is complex for general asymmetric *ω*_**k**_, for example in inversion-asymmetric environments. We observe that both local and nonlocal spectral densities encompass contributions from environmental excitations with finite wavelengths. This effectively captures information about the correlations of environment.

It is worth noting that the noise power spectral density (9) is generally asymmetric in frequency, ∣*S*_*ij*_(*ω*)∣ ≠ ∣*S*_*ji*_( − *ω*)∣. Its positive- and negative-frequency components are linked through the Boltzmann factor, *S*_*ij*_(*ω*) = *e*^*βℏω*^*S*_*ji*_( − *ω*), which reflects the quantum nature of the noise, as indicated by the non-zero commutator [*E*_1_(*t*), *E*_2_] ≠ 0. One can interpret the positive-frequency part *S*_*ij*_(*ω* > 0) as a measure of the ability of qubits to emit energy, and the negative-frequency part *S*_*ij*_(*ω* < 0) as a measure of the ability of qubits to absorb energy.

When *E* is a classical field, namely [*E*_*i*_(*t*), *E*_*j*_] = 0, the associated noise is referred to as classical noise. In the case of a quantum field *E*, we can write the noise correlation function *S*_*ij*_(*t*) as a sum of symmetrized [invariant under the exchange of *E*_*i*_(*t*) and *E*_*j*_] and antisymmetrized parts, which are identified as classical and quantum noise spectral densities respectively, following the definition in refs. ^8,83^:

10 $${S}_{ij}^{C}(\omega )=\frac{{S}_{ij}(\omega )+{S}_{ji}(-\omega )}{2},\,{S}_{ij}^{Q}(\omega )=\frac{{S}_{ij}(\omega )-{S}_{ji}(-\omega )}{2}.$$

They are related to each other through ${S}_{ij}^{C}(\omega )=\coth (\beta \hslash \omega /2){S}_{ij}^{Q}(\omega )$. It is dictated by the fluctuation-dissipation theorem^84^, which holds when the average of the noise correlator is taken with respect to the thermal state *ρ*_B_ of the environment. We also point out that this definition is operationally meaningful. As demonstrated in our later discussions, correlated classical noise does not result in entanglement between qubits, whereas quantum noise effectively induces entanglement.

When operating qubits at high temperatures, such as in the case of spin qubits that can be operated at a few Kelvin^82,85–87^, the classical limit *k*_B_*T* ≫ *ℏω* applies, and classical fluctuations dominate over quantum noise. In this regime, the operators *E*_*i*_(*t*) can be treated classically [*E*_*i*_(*t*), *E*_*j*_(0)] = 0, resulting in a symmetric noise spectral density with a vanishing antisymmetric part, *S*^*Q*^ = 0. In contrast, in the quantum regime where *k*_B_*T* ≲ *ℏω*, the quantum noise is comparable to classical noise, with *S*^*Q*^ ≈ *S*^*C*^, and therefore cannot be neglected.

**Fig. 2:**
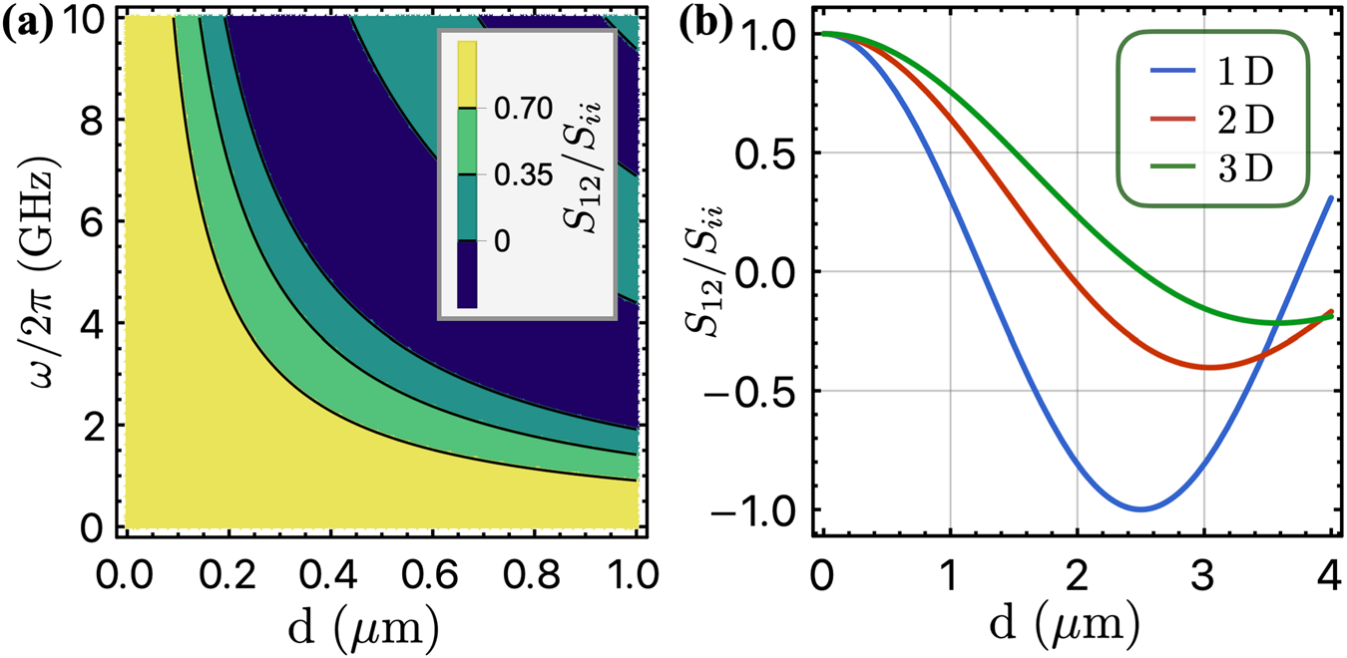
Correlated noise. **a** Density plot of the ratio between the spatially correlated noise *S*_12_ and the local noise *S*_*ii*_ as a function of frequency *ω* and the distance *d* between two qubits in two dimensions. The plot shows that for qubit frequencies in the few GHz regime, the correlated noise is as strong as the local one when two qubits sit within micrometers, and it oscillates and decays to zero as the distance increases. **b** The ratio *S*_12_/*S*_*ii*_ as a function of qubit distance in different dimensions, where we have taken *ω*/2*π* = 1GHz. The correlated noise exhibits similar behavior in different dimensions, and obeys the constraint *S*_12_ ≤ *S*_*ii*_. In both plots, we used *ω*_**k**_ = *c*_*s*_∣**k**∣ and *c*_*s*_ = 5 km/s.

We point out that there is a constraint for the spatially correlated noise, $| {S}_{12}^{C}(\omega ){| }^{2}\le {S}_{11}^{C}(\omega ){S}_{22}^{C}(\omega )$^8^, implying that the nonlocal noise is inherently bounded by the local one. This condition is closely related to the thermodynamic stability of the environment^65^. To illustrate this condition explicitly, we consider a concrete example, where the environment has a linear spectrum *ω*_**k**_ = *c*_*s*_∣**k**∣. This case describes for example acoustic phonons with sound velocity *c*_*s*_. The spatially correlated noise is related to the local noise in a 2D architecture through the following equation:

11 $${S}_{12}(\omega )={J}_{0}(\omega d/{c}_{s}){S}_{ii}(\omega ),$$

where ∣*J*_0_(*x*)∣ ≤ 1 is the Bessel functions of the first kind and *d* = ∣**r**_1_ − **r**_2_∣ is the distance between two qubits, as shown in Fig. 2a. At large distances, the correlated noise decays as ${J}_{0}(\omega d/{c}_{s}) \sim 1/\sqrt{d}$. We will assume the two qubits to be identical and thus they experience the same local noise *S*_11_ = *S*_22_ throughout our discussion. The constraint ∣*S*_12_∣ ≤ *S*_*ii*_ also holds in other dimensions, as shown in Fig. 2b where the ratio *S*_12_/*S*_*ii*_ is shown as a function of the separation between the two qubits in different dimensions. Exact relations are provided in Supplementary Note 1.

We remark that, when the two qubits are sitting within a few hundred nanometers, the spatially correlated noise is comparable to the local noise for qubit frequencies in the gigahertz range, as illustrated in Fig. 2a, and assuming the sound velocity to be *c*_*s*_ ~ 5 km/s in silicon. The correlated noise exhibits oscillatory behavior and gradually decays to zero as the qubit separation *d* increases. As shown in Fig. 2b where we take the frequency to be *ω*/2*π* = 1 GHz, the spatially correlated noise is always comparable with the local noise for *d* < 1 *μ*m in different dimensions.

### Time convolutionless master equation

We now extend the commonly used master equation for qubit dynamics to a set of broadly applicable equations for qubits subjected to spatially correlated classical and quantum noise. The master equation for the reduced density matrix *ρ*(*t*) is obtained by tracing out the environment from the total density matrix *ρ*_tot_(*t*). To this end, we adopt the time-convolutionless (TCL) master equation approach^88^ and assume that the qubit-environment interaction is weak enough to truncate the TCL generator at the second order. Leaving the detailed derivation to Supplementary Note 2, here we present the TCL master equations for the two-qubit system subject to correlated noise, without and with a resonant drive, respectively. In particular, we separate the quantum and classical noise, enabling us to clearly identify their respective contributions to the qubit dynamics. Our results allow us to explicitly calculate and explore the effects of correlated noise.

In the absence of coherent driving, the qubit dynamics is purely determined by dephasing. As detailed in Supplementary Note 2, the TCL master equation for the two-qubit system, in the interaction picture, takes the form of

12 $$\dot{\rho }(t)=-i[{H}_{z}(t),\rho (t)]+\mathop{\sum}\limits_{i,j\in \{1,2\}}{{{{\mathcal{L}}}}}_{ij}^{z}(t)\rho (t),$$

where the correlated-noise-induced coherent interaction between qubits is Ising-like:

13 $${H}_{z}(t)={{{{\mathcal{J}}}}}^{z}(t){\sigma }_{1}^{z}{\sigma }_{2}^{z}.$$

The superoperators ${{{{\mathcal{L}}}}}_{ij}^{z}(t)$ are defined by

14 $${{{{\mathcal{L}}}}}_{ij}^{z}(t)\rho ={\gamma }_{ij}^{z}(t)\left[{\sigma }_{j}^{z}\rho {\sigma }_{i}^{z}-\frac{1}{2}\{{\sigma }_{i}^{z}{\sigma }_{j}^{z},\rho \}\right],$$

with the anticommutator {*A*, *B*} ≡ *AB* + *BA*. Here, ${\gamma }_{ii}^{z}(t)$ stands for the standard local dephasing which is time-dependent in general, whereas ${\gamma }_{12}^{z}(t)$ represents correlated dephasing originating from the spatially correlated noise. The time-dependent coherent coupling parameter is given by ${{{{\mathcal{J}}}}}^{z}(t)=\int\nolimits_{0}^{t}ds\left[{{{{\mathcal{G}}}}}_{12}^{R}(s)+{{{{\mathcal{G}}}}}_{21}^{R}(s)\right]/2{\hslash }^{2}$, quantifying the retarded interaction mediated by the environment, where ${{{{\mathcal{G}}}}}_{12}^{R}(t)=-i\Theta (t)\langle [{E}_{1}(t),{E}_{2}]\rangle$ is the standard retarded Green’s function. To clarify its relation to the correlated noise, it is insightful to recast it into the following form:

15 $${{{{\mathcal{J}}}}}^{z}(t)=\frac{4}{{\hslash }^{2}}\int\nolimits_{0}^{\infty }\frac{d\omega }{2\pi }{{{\rm{Re}}}}[{S}_{12}^{Q}(\omega )]{F}_{c}(\omega ,t),$$

with a filter function ${F}_{c}(\omega ,t)=[\cos (\omega t)-1]/\omega$. This coherent Ising interaction is solely determined by the correlated quantum noise. One can also infer this conclusion from the fact that it is dictated by the retarded Green’s function which vanishes when the noise operators *E*_*i*_ commute, e.g., if they are classical variables.

Similarly, to investigate the effects of correlated classical and quantum noise on the dephasing, we write the dephasing rate as

16 $${\gamma }_{ij}^{z}(t)=\frac{4}{{\hslash }^{2}}\int\nolimits_{0}^{\infty }\frac{d\omega }{2\pi }{F}_{s}(\omega ,t)\left\{{{{\rm{Re}}}}[{S}_{ij}^{C}(\omega )]+i\,{{{\rm{Im}}}}[{S}_{ij}^{Q}(\omega )]\right\},$$

with the filter function ${F}_{s}(\omega ,t)=\sin \omega t/\omega$, which is peaked at zero frequency and approaches a delta function at large time *F*_*s*_(*ω*, *t* → *∞*) = *πδ*(*ω*). We observe that the local dephasing rate ${\gamma }_{ii}^{z}$ is determined solely by the classical noise ${S}_{ii}^{C}$ (where we recall that the local noise spectral densities ${S}_{ii}^{C,Q}$ are always real), whereas the correlated dephasing ${\gamma }_{12}^{z}$ has contributions from both classical and quantum spatially correlated noise. In contrast, we stress again that the coherent coupling is not affected by classical noise. Furthermore, it is worth noting that correlated quantum noise only influences the dephasing process when the environment has an asymmetric spectrum such that ${{{\rm{Im}}}}[{S}_{12}^{Q}]\,\ne\, 0$. Otherwise, ${\gamma }_{12}^{z}(t)$ is exclusively determined by the correlated classical noise ${S}_{12}^{C}(\omega )$.

In the presence of resonant transverse drives, the combined system is governed by the Hamiltonian (5) where the qubits experience pure transverse noise. The TCL master equation of the two-qubit system is shown to be the following form

17 $$\dot{\rho }(t)=-i[{H}_{xy}(t),\rho (t)]+\mathop{\sum}\limits_{i,j\in \{1,2\}}{{{{\mathcal{L}}}}}_{ij}(t)\rho (t),$$

where the coherent interaction is

18 $${H}_{xy}(t)=\,{{{\mathcal{J}}}}(t){\hat{\sigma }}_{1}^{+}{\hat{\sigma }}_{2}^{-}+\,\,{{\mbox{H.c.}}}\,,$$

and the superoperators are given by

19 $$\begin{array}{ll}{{{{\mathcal{L}}}}}_{ij}(t)\rho\,=\,{\gamma }_{ij}^{\downarrow }(t)\left[{\hat{\sigma }}_{j}^{-}\rho {\hat{\sigma }}_{i}^{+}-\frac{1}{2}\{{\hat{\sigma }}_{i}^{+}{\hat{\sigma }}_{j}^{-},\rho \}\right]\\\qquad\qquad+\,{\gamma }_{ij}^{\uparrow }(t)\left[{\hat{\sigma }}_{j}^{+}\rho {\hat{\sigma }}_{i}^{-}-\frac{1}{2}\{{\hat{\sigma }}_{i}^{-}{\hat{\sigma }}_{j}^{+},\rho \}\right].\end{array}$$

Here ${\gamma }_{ij}^{\downarrow }$ and ${\gamma }_{ij}^{\uparrow }$ describe time-dependent (local and correlated) decay and absorption rates, respectively. Detailed derivations are provided in Supplementary Note 2. Similar to the pure dephasing case, the coupling parameter ${{{\mathcal{J}}}}(t)$ is also fully determined by the quantum noise, and can be expressed in terms of the retarded Green’s functions or correlated noise spectral densities as

20 $$\begin{array}{ll}{{{\mathcal{J}}}}(t)\,=\,\frac{1}{2{\hslash }^{2}}\int\nolimits_{0}^{t}ds\left[{{{{\mathcal{G}}}}}_{12}^{R}{e}^{i\Omega s}+{{{{\mathcal{G}}}}}_{21}^{R}(s){e}^{-i\Omega s}\right]\\\quad\quad\,\,\,=\,\frac{2}{{\hslash }^{2}}\int\nolimits_{0}^{\infty }\frac{d\omega }{2\pi }\left[{S}_{12}^{Q}(\omega ){F}_{c}(\omega -\Omega ,t)+{S}_{21}^{Q}(\omega ){F}_{c}(\omega +\Omega ,t)\right],\end{array}$$

with qubit energy splitting Ω. We stress that, in contrast to the Ising coupling ${{{{\mathcal{J}}}}}^{z}(t)$ which is always real, ${{{\mathcal{J}}}}(t)$ is complex in general.

The local and correlated decay processes are induced by both classical and quantum noise, ${\gamma }_{ij}^{\downarrow }(t)={\gamma }_{ij}^{C}(t,\Omega )+{\gamma }_{ij}^{Q}(t,\Omega )$, with classical and quantum contributions being

21 $$\begin{array}{l}{\gamma }_{ij}^{C}(t,\Omega )\,=\,\frac{2}{{\hslash }^{2}}\int\nolimits_{0}^{\infty }\frac{d\omega }{2\pi }\left[{S}_{ij}^{C}(\omega ){F}_{s}(\omega -\Omega ,t)+{S}_{ji}^{C}(\omega ){F}_{s}(\omega +\Omega ,t)\right],\\ {\gamma }_{ij}^{Q}(t,\Omega )\,=\,\frac{2}{{\hslash }^{2}}\int\nolimits_{0}^{\infty }\frac{d\omega }{2\pi }\left[{S}_{ij}^{Q}(\omega ){F}_{s}(\omega -\Omega ,t)-{S}_{ji}^{Q}(\omega ){F}_{s}(\omega +\Omega ,t)\right].\end{array}$$

Similarly, the local and correlated absorption rates are given by ${\gamma }_{ij}^{\uparrow }(t)={\gamma }_{ij}^{C}(t,-\Omega )+{\gamma }_{ij}^{Q}(t,-\Omega )$. Notably, unlike the local pure dephasing rate that is solely determined by classical noise, ${\gamma }_{ii}^{\uparrow ,\downarrow }$ depends on both classical and quantum noise. Moreover, the correlated quantum noise is always present in the correlated decay and absorption rates, regardless of the symmetry of the spectrum *ω*_**k**_. We emphasize that these processes exhibit high sensitivity to the noise spectra within a frequency window of approximately 1/*t* centered on the qubit splitting ± Ω. Therefore, for sufficiently long time evolution where *t* ≫ 1/Ω, we can consider exclusively the contribution of the spectra at *ω* = ± Ω, and approximate the rates as

22 $${\gamma }_{ij}^{\downarrow }=\frac{1}{{\hslash }^{2}}\left[{S}_{ij}^{C}(\Omega )+{S}_{ij}^{Q}(\Omega )\right],\quad {\gamma }_{ij}^{\uparrow }=\frac{1}{{\hslash }^{2}}\left[{S}_{ji}^{C}(\Omega )-{S}_{ji}^{Q}(\Omega )\right],$$

from where we observe that the asymmetry between the absorption and emission is caused by quantum noise. We also obtain the standard detailed balance condition ${\gamma }_{ij}^{\downarrow }={e}^{\beta \hslash \Omega }{\gamma }_{ji}^{\uparrow }$, by invoking the fluctuation-dissipation theorem.

The TCL master equations presented here for the two-qubit system show clearly the dependence of qubit dynamics on the quantum and classical components of local and spatially correlated noise. Our formalism is general, and it does not require any microscopic understanding of the spatial and temporal correlations within the environment, as long as the noise is weak enough to justify the truncation of the TCL generator at the second order. It can be employed to describe challenging cases such as long-ranged classical or quantum non-Markovian noise. Furthermore, this approach can be extended straightforwardly to multiple qubits. By separating the contributions of classical and quantum noise to the qubit dynamics, this scheme provides a foundation for our investigation of the impact of generic noise on multiqubit dynamics.

### Idle Qubits subjected to correlated 1/*f* noise

We now utilize the formalism presented above to investigate the impact of spatially correlated classical and quantum noise on the dephasing of two qubits. Specifically, we focus on a noise spectral function with a 1/*f* frequency dependence, which is common in various quantum computing architectures^23^, including superconducting qubits and semiconducting devices. We stress that our approach is straightforwardly extended to other noise spectra. Let us consider a local classical noise spectral density:

23 $${S}_{ii}^{C}(\omega )=\left\{\begin{array}{ll}2\pi {\sigma }^{2}/| \omega | ,\quad &{{{\rm{if}}}}\,| \omega | > {\omega }_{l},\\ \,\,0,\quad &{{{\rm{otherwise}}}},\end{array}\right.$$

where *σ* is the standard derivation of the noise and *ω*_*l*_ stands for the low frequency cutoff that is set by the measurement time. The timescales that we investigate are much shorter than this time, $t\ll {\omega }_{l}^{-1}$. As we aim to study the effect of the correlated noise, we consider two qubits positioned within the range of micrometers, with correlated noise being comparable with the local one, ${S}_{12}^{C,Q}(\omega )\approx {e}^{i\theta }{S}_{ii}^{C,Q}(\omega )$, where *θ* is the phase of the correlated noise spectral density that characterizes its complex nature (The phase *θ* has a frequency dependence in general whose exact form depends on the details of the environment and the coupling. In this work, we assume the phase to be a constant for simplicity.). We point that in our study, we assume that the distance between the two qubits is greater than the typical confinement lengths (10–50 nm for spin qubits). This ensures that the direct exchange interaction is suppressed, allowing us to focus specifically on the effects induced by correlated noise.

To examine how the classical and quantum components of the spatially correlated noise determine the two-qubit dynamics, we consider the quantum regime, where both quantum and classical noises are present and *S*^*Q*^ ≈ *S*^*C*^. In this scenario, the coherent coupling and the local dephasing rate ${\gamma }^{z}\equiv {\gamma }_{ii}^{z}$ are given by

24 $${{{{\mathcal{J}}}}}^{z}(t)=-\frac{2\pi {\sigma }^{2}\cos \theta }{{\hslash }^{2}}t,\,\,{\gamma }^{z}(t)=\frac{4{\sigma }^{2}t}{{\hslash }^{2}}\left[1-{{{\rm{Ci}}}}({\omega }_{l}t)\right],$$

as detailed in Supplementary Note 3, and the correlated dephasing rate is given by ${\gamma }_{12}^{z}={e}^{i\theta }{\gamma }^{z}$ whose real and imaginary parts are rooted in classical and quantum correlated noise, respectively. Here, ${{{\rm{Ci}}}}(x)\equiv -\int\nolimits_{x}^{\infty }d\tau \,\cos (\tau )/\tau$ is the cosine integral function.

It is convenient to work in the basis $\{\left\vert a\right\rangle \}=\{\left\vert \uparrow \uparrow \right\rangle ,\left\vert \uparrow \downarrow \right\rangle ,\left\vert \downarrow \uparrow \right\rangle ,\left\vert \downarrow \downarrow \right\rangle \}$, where the density matrix elements, denoted as $\rho ={G}_{ab}\left\vert a\right\rangle \left\langle b\right\vert$, are all decoupled from each other. While the diagonal elements *G*_*aa*_ remain constant in the pure-dephasing dynamics, the off-diagonal components depend non-trivially on the correlated noise. By solving the TCL master equation analytically (see Supplementary Note 3 for details), we find that the classical component of the correlated noise can only reduce or enhance the dephasing rate caused by the local classical noise, without increasing the coherence in the two-qubit system. For instance, *G*_23_ and *G*_14_ are given by ${G}_{23}(t)={G}_{23}(0)\exp [-4(1-\cos \theta ){\Gamma }^{z}(t)]$ and ${G}_{14}(t)={G}_{14}(0)\exp [-4(1+\cos \theta ){\Gamma }^{z}(t)]$ with

25 $${\Gamma }^{z}(t)\equiv \int\nolimits_{0}^{t}ds\,{\gamma }^{z}(s)\approx \frac{{\sigma }^{2}{t}^{2}}{{\hslash }^{2}}\left[3-2\gamma -2\ln ({\omega }_{l}t)\right],$$

suggesting a Gaussian decay of the coherence with a logarithmic correction. Here, *γ* is Euler’s constant.

**Fig. 3:**
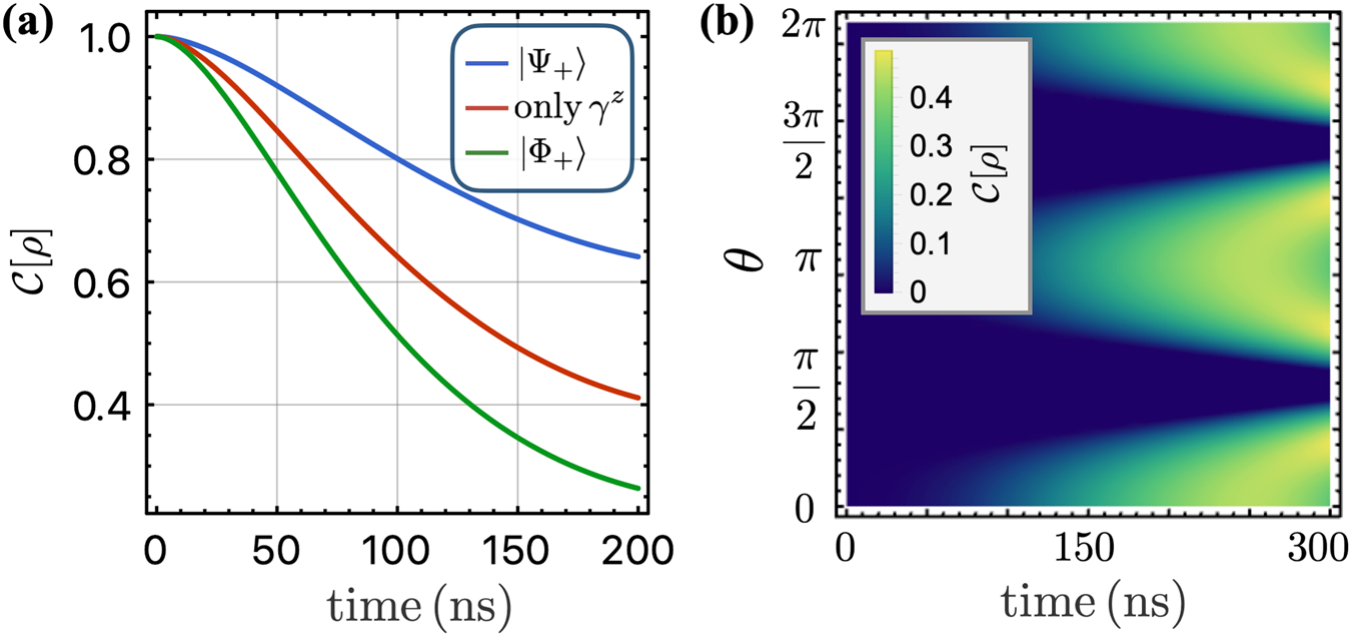
Dephasing of two qubits subjected to spatially correlated noise. **a** Entanglement decay as a function of time for different initial states. The red curve corresponds to the case where only local noise is present, showing that both scenarios [with initial states being Bell states $\left\vert {\Psi }_{+}\right\rangle =(\left\vert \uparrow \downarrow \right\rangle +\left\vert \downarrow \uparrow \right\rangle )/\sqrt{2}$ and $\left\vert {\Phi }_{+}\right\rangle =(\left\vert \uparrow \uparrow \right\rangle +\left\vert \downarrow \downarrow \right\rangle )/\sqrt{2}$] decay at the same rates. The blue and green curves show the entanglement decay with initial states $\left\vert {\Psi }_{+}\right\rangle$ and $\left\vert {\Phi }_{+}\right\rangle$, respectively, in the presence of correlated classical noise. We set the quantum noise to zero and choose a phase of *θ* = *π*/3 for the correlated noise power spectral density *S*_12_(*ω*). **b** Density plot of the entanglement as a function of time and the angle *θ* with initial state $\left\vert ++\right\rangle$ ($\left\vert +\right\rangle$ is defined by ${\sigma }^{x}\left\vert +\right\rangle =\left\vert +\right\rangle$). The correlated quantum noise is real and enters the qubits dynamics through the coherent coupling when *θ* = 0, *π*, and is purely imaginary and enters the dynamics through the correlated dephasing when *θ* = *π*/2, 3*π*/2. The correlated quantum noise does not generate entanglement through the correlated dephasing, but it generates it via the noise-induced Ising coherent coupling ${{{{\mathcal{J}}}}}^{z}$. Parameters used in the plot: *ℏ*/*σ* = 500 ns and *ω*_*l*_/2*π* = 1 MHz.

We are now ready to investigate how the correlated noise affects the dynamics of two-qubit entanglement. There are different measures of entanglement. For example, the singlet fidelity of the corresponding Werner state^89^ of an arbitrary mixed state provides a lower bound for the entanglement of formation^90,91^, as detailed in Supplementary Note 4. In this work, we adopt the two-qubit concurrence as a measure of entanglement^92^, which is also summarized in Supplementary Note 4. In Fig. 3a, we illustrate the entanglement decay of the two-qubit system with the initial states being Bell states $\left\vert {\Psi }_{+}\right\rangle \equiv (\left\vert \uparrow \downarrow \right\rangle +\left\vert \downarrow \uparrow \right\rangle )/\sqrt{2}$ and $\left\vert {\Phi }_{+}\right\rangle \equiv (\left\vert \uparrow \uparrow \right\rangle +\left\vert \downarrow \downarrow \right\rangle )/\sqrt{2}$, respectively. The red curve represents the entanglement decay when only local noise is present, where both states decay with the same rates. When the correlated classical noise is present (we set the quantum noise to zero and *θ* = *π*/3), both states still decay but with different rates, corresponding to the blue and green curve in Fig. 3a.

The quantum component of the correlated noise affects the two-qubit dynamics through both the coherent Ising interaction, which is governed by the real part of ${S}_{12}^{Q}$, and the correlated dephasing, which is linked to the imaginary part of ${S}_{12}^{Q}$. Interestingly, we find that only the real part of the quantum correlated noise leads to an increase in the entanglement between the two qubits. To illustrate this effect, we consider an initial state $\left\vert ++\right\rangle$, where $\left\vert +\right\rangle$ is defined by ${\sigma }^{x}\left\vert +\right\rangle =\left\vert +\right\rangle$. We examine the entanglement dynamics as a function of time and phase *θ*. When the spectral density is real (*θ* = 0, *π*), we observe a sizable increase in entanglement, while it remains at zero when the spectral density is purely imaginary (*θ* = *π*/2, 3*π*/2), as shown in Fig. 3b.

Our findings indicate that to harness the correlations encoded in the pure-dephasing noise, qubits must be operated at low temperatures to ensure the presence of quantum correlated noise and in an inversion symmetric environment to obtain a real quantum noise spectral density. Conversely, if one wishes to prevent undesired entanglement between the qubits, operating the qubits at higher temperatures to favor classical correlated noise or breaking the symmetry of the environment at low temperatures to suppress entanglement generation can be effective strategies. We notice that a very recent experiment^66^ has observed an unexpected significant reduction in crosstalk between spin qubits at higher temperatures in semiconductors.

### Driven spin qubits subject to correlated Markovian noise

Here, we investigate the dynamics of two qubits when they are resonantly driven. This case is governed by Hamiltonian (5), and the reduced dynamics is described by the master equation (17). In particular, we focus on the temporally uncorrelated (Markovian) noise, because this model accurately describes noise with generic power spectral density after long times. As detailed in Supplementary Note 4, pure classical noise does not generate entanglement, hence we examine the case of low temperature where both classical and quantum noise are present. With the spatially correlated Markovian noise, the coherent interaction between the two qubits becomes time-independent, and is described by the Hamiltonian

26 $${H}_{xy}={{{\mathcal{J}}}}{\hat{\sigma }}_{1}^{+}{\hat{\sigma }}_{2}^{-}+{{{{\mathcal{J}}}}}^{* }{\hat{\sigma }}_{1}^{-}{\hat{\sigma }}_{2}^{+}.$$

The coupling strength

27 $${{{\mathcal{J}}}}=\frac{{{{{\mathcal{G}}}}}_{12}^{R}(\Omega )+{{{{\mathcal{G}}}}}_{21}^{R}(-\Omega )}{2{\hslash }^{2}}$$

is complex-valued in general and denoted as ${{{\mathcal{J}}}}\equiv {{{{\mathcal{J}}}}}_{s}+i{{\mathcal{{D}}}}$. The contributions ${{{{\mathcal{J}}}}}_{s}$ and Dzyaloshinskii-Moriya (DM) interaction ${{{\mathcal{D}}}}$ are symmetric and antisymmetric with respect to exchange of the two qubits, respectively,

28 $${H}_{xy}={{{{\mathcal{J}}}}}_{s}({\hat{\sigma }}_{1}^{x}{\hat{\sigma }}_{2}^{x}+{\hat{\sigma }}_{1}^{y}{\hat{\sigma }}_{2}^{y})+{{{\mathcal{D}}}}\hat{z}\cdot {\overrightarrow{\hat{\sigma }}}_{1}\times {\overrightarrow{\hat{\sigma }}}_{2},$$

where the DM interaction only arises when the inversion symmetry of the environment is broken.

The decay and absorption rates become time-independent when noise is assumed to be Markovian (or when the evolution time is long for generic noise, *t* ≫ 1/Ω) and are given by Eq. (22). At temperatures lower than the Rabi frequency, *k*_*B*_*T*≤*ℏ*Ω, (for instance, when Ω/2*π* ~ 2 GHz and the temperature is below 100 mK), the rates are further reduced to ${\gamma }_{ij}^{\downarrow }\approx 2{S}_{ij}^{Q}(\Omega )$ and ${\gamma }_{ij}^{\uparrow }\approx 0$. In this situation, the superoperators are given by the standard Lindbladians^88^:

29 $$\begin{array}{ll}{{{{\mathcal{L}}}}}_{ij}\rho\,=\,{\gamma }^{\downarrow }\mathop{\sum}\limits_{i}\left[{\hat{\sigma }}_{i}^{-}\rho {\hat{\sigma }}_{i}^{+}-\frac{1}{2}\{{\hat{\sigma }}_{i}^{+}{\hat{\sigma }}_{i}^{-},\rho \}\right]\\\quad\quad\,\,+\,{\gamma }_{12}^{\downarrow }\mathop{\sum}\limits_{i\ne j}\left[{\hat{\sigma }}_{j}^{-}\rho {\hat{\sigma }}_{i}^{+}-\frac{1}{2}\{{\hat{\sigma }}_{i}^{+}{\hat{\sigma }}_{j}^{-},\rho \}\right],\end{array}$$

where the local decay rate ${\gamma }^{\downarrow }\equiv {\gamma }_{ii}^{\downarrow }$ and the collective decay rate ${\gamma }_{12}^{\downarrow } > 0$ are determined by the local and spatially correlated noise, respectively. We have absorbed the phase of ${\gamma }_{12}^{\downarrow }$ into the definition of ${\sigma }_{i}^{\pm }$. The completely positive evolution dictates that the correlated decay is weaker than the local decay ${\gamma }_{12}^{\downarrow }\le {\gamma }^{\downarrow }$, which is also guaranteed by the thermodynamic stability of the environment^65^.

Similar to the coherent interaction, it is convenient to symmetrize and antisymmetrize the superoperators, yielding

30 $${{{\mathcal{L}}}}\rho =\mathop{\sum}\limits_{\alpha \in \{S,A\}}{\Gamma }_{\alpha }\left[{\hat{\sigma }}_{\alpha }^{-}\rho {\hat{\sigma }}_{\alpha }^{+}-\frac{1}{2}\{{\hat{\sigma }}_{\alpha }^{+}{\hat{\sigma }}_{\alpha }^{-},\rho \}\right],$$

where ${\sigma }_{S,A}^{+}=({\sigma }_{1}^{+}\pm {\sigma }_{2}^{+})/\sqrt{2}$ and ${\Gamma }_{S,A}={\gamma }^{\downarrow }\pm {\gamma }_{12}^{\downarrow }$. We note that the triplet state $\left\vert T\right\rangle \equiv (\left\vert \uparrow \downarrow \right\rangle +\left\vert \downarrow \uparrow \right\rangle )/\sqrt{2}$ is superradiant decaying at rate Γ_*S*_, while the singlet state, $\left\vert S\right\rangle \equiv (\left\vert \uparrow \downarrow \right\rangle -\left\vert \downarrow \uparrow \right\rangle )/\sqrt{2}$ is subradiant decaying at rate Γ_*A*_. We also remark that these two states are decoupled from each other in ${{{\mathcal{L}}}}\rho$, and are also eigenstates of the symmetric interaction characterized by ${{{{\mathcal{J}}}}}_{s}$. However, the DM interaction, which is parity-odd, exchanges these two states $(\hat{z}\cdot {\overrightarrow{\hat{\sigma }}}_{1}\times {\overrightarrow{\hat{\sigma }}}_{2})\left\vert T,S\right\rangle \propto \left\vert S,T\right\rangle$.

In the following subsection, we analytically study the interplay between the symmetric interaction, the DM interaction, and the local and correlated decay processes. For the sake of concreteness, we assume the initial state is a trivial product state $\left\vert \uparrow \downarrow \right\rangle$. We denote the density matrix as $\rho ={G}_{t}\left\vert T\right\rangle \left\langle T\right\vert +{G}_{s}\left\vert S\right\rangle \left\langle S\right\vert +\left({G}_{ts}\left\vert T\right\rangle \left\langle S\right\vert +\,{{\mbox{H.c.}}}\,\right)+\Delta \rho$, where Δ*ρ* stands for other elements of the density matrix. In this scenario, the concurrence of the two-qubit system can be shown to take the simple form of ${{{\mathcal{C}}}}[\rho ]=| {{{{\mathcal{C}}}}}_{R}+i{{{{\mathcal{C}}}}}_{I}|$, with

31 $${{{{\mathcal{C}}}}}_{R}\equiv {G}_{s}-{G}_{t},\quad {{{\rm{and}}}}\quad {{{{\mathcal{C}}}}}_{I}\equiv 2\,{{{\rm{Im}}}}\,{G}_{ts},$$

which indicates that the entanglement of the qubits has two independent contributions (see Supplementary Note 4 for detailed derivations). To gain some physical understanding of this expression, we observe that the first contribution arises from the asymmetry in the populations of the triplet state $\left\vert T\right\rangle$ and singlet state $\left\vert S\right\rangle$, which is proportional to $\left\vert \uparrow \downarrow \right\rangle \left\langle \downarrow \uparrow \right\vert +\left\vert \downarrow \uparrow \right\rangle \left\langle \uparrow \downarrow \right\vert$. The second contribution comes from a finite imaginary part of *G*_*ts*_, which is proportional to $\left\vert \uparrow \downarrow \right\rangle \left\langle \downarrow \uparrow \right\vert -\left\vert \downarrow \uparrow \right\rangle \left\langle \uparrow \downarrow \right\vert$. Both indeed characterize the coherence (superposition) of $\left\vert \uparrow \downarrow \right\rangle$ and $\left\vert \downarrow \uparrow \right\rangle$.

**Fig. 4:**
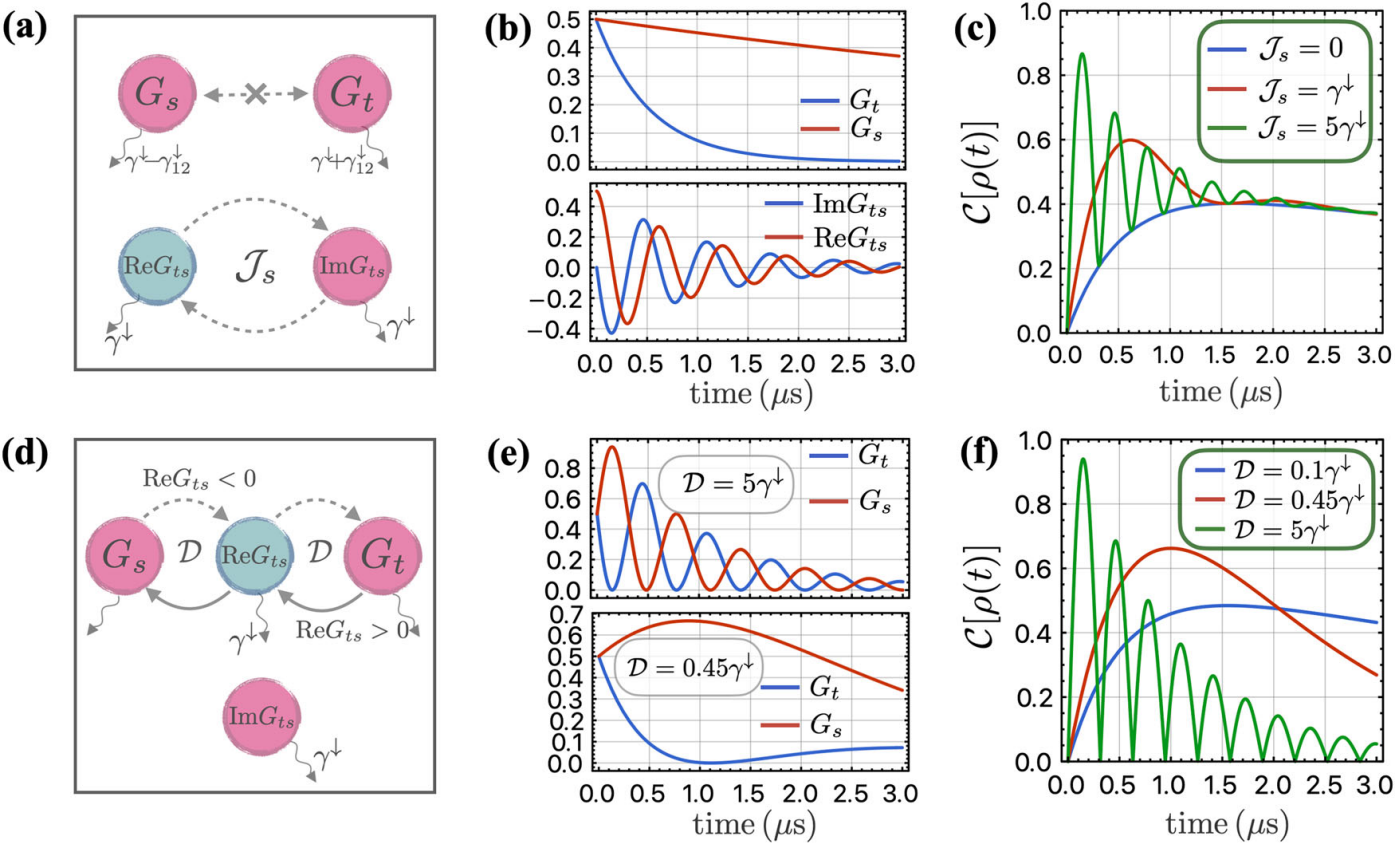
Entanglement dynamics of resonantly driven qubits with initial state $\left\vert \uparrow \downarrow \right\rangle$. **a**–**c** Qubit dynamics in the absence of DM interaction ${{{\mathcal{D}}}}$. **a** Schematic for the coupled dynamics of relevant density matrix elements. In the absence of the DM interaction ${{{\mathcal{D}}}}=0$, the dynamics of *G*_*s*_ and *G*_*t*_ are decoupled from each other, decaying independently at rates of ${\gamma }^{\downarrow }-{\gamma }_{12}^{\downarrow }$ and ${\gamma }^{\downarrow }+{\gamma }_{12}^{\downarrow }$, respectively. The real and imaginary parts of *G*_*ts*_ are coupled to each other through the symmetric coupling ${{{{\mathcal{J}}}}}_{s}$, while they decay at the same rate *γ*^*↓*^. **b** The upper panel illustrates the decay of the superradiant state $\left\vert T\right\rangle$ and the subradiant state $\left\vert S\right\rangle$, which is independent of the parameter ${{{{\mathcal{J}}}}}_{s}$. The lower panel shows the oscillations of the real and imaginary parts of *G*_*ts*_ with frequency $2{{{{\mathcal{J}}}}}_{s}$, where we use parameter ${{{{\mathcal{J}}}}}_{s}=5{\gamma }^{\downarrow }$. **c** Entanglement quantified by the concurrence ${{{\mathcal{C}}}}[\rho (t)]$ between two qubits as a function of time with varying parameter ${{{{\mathcal{J}}}}}_{s}$. The oscillation frequency of the entanglement is $\propto {{{{\mathcal{J}}}}}_{s}$. The entanglement is bounded below by ∣*G*_*s*_ − *G*_*t*_∣ at any time. At large time *t* ≫ 1/*γ*^*↓*^, the oscillation is insignificant and the dynamics is dominated by the local and correlated noise. **d**–**f** Qubit dynamics in the absence of symmetric interaction. **d** The dynamics of *G*_*t*_ and *G*_*s*_ are coupled to each other via ${{{\rm{Re}}}}\,{G}_{ts}$, in the presence of the DM interaction, while ${{{\rm{Im}}}}\,{G}_{ts}$ is decoupled from other elements. Assuming ${{{\mathcal{D}}}} \,>\, 0$ without loss of generality, the probability in $\left\vert S\right\rangle$ would flow to *G*_*t*_ when ${{{\rm{Re}}}}\,{G}_{ts} < 0$, whereas the probability flows in the opposite direction when ${{{\rm{Re}}}}\,{G}_{ts} \,>\, 0$. The changing rate of ${{{\rm{Re}}}}\,{G}_{ts}$ is determined by the difference between *G*_*t*_ and *G*_*s*_, ${{{\rm{Re}}}}{\partial }_{t}{G}_{ts}=-{\gamma }^{\downarrow }{{{\rm{Re}}}}\,{G}_{ts}+{{{\mathcal{D}}}}({G}_{t}-{G}_{s})$. **e** The upper panel shows the oscillations of *G*_*t*_ and *G*_*s*_ when ${{{\mathcal{D}}}}=5{\gamma }^{\downarrow }$. The lower panel shows the their time evolution when the DM coupling is small ${{{\mathcal{D}}}}=0.45{\gamma }^{\downarrow }$, where we do not see the oscillation behavior as the dynamics is overdamped. **f** Entanglement between two qubits as a function of time with varying strength of DM coupling ${{{\mathcal{D}}}}$. The green curve shows the oscillation of entanglement, where the DM coupling is large and the dynamics is underdamped.The red curve is the critical point, where the entanglement stops oscillating and behaves as $\propto t{e}^{-{\gamma }^{\downarrow }t}$. The blue curve is when the DM coupling is small, where the dynamics is overdamped. Parameters used in all figures: *γ*^*↓*^ = 1 *μ*s^−1^ and ${\gamma }_{12}^{\downarrow }=0.9{\gamma }^{\downarrow }$.

Le us now analyze one basic scenario in which the environment possesses inversion symmetry, resulting in a real correlated noise spectral density *S*_12_(*ω*). In this case, the DM interaction is absent. The dynamics of the singlet state *G*_*s*_ and triplet state *G*_*t*_ are decoupled due to the symmetry, while the real and imaginary parts of *G*_*ts*_ are coupled to each other due to the symmetric exchange coupling ${{{{\mathcal{J}}}}}_{s}$, as illustrated in Fig. 4a. The complete dynamics is analytically solved in the Supplementary Note 4.

The impact of correlated quantum noise on the two-qubit dynamics is twofold. Firstly, the noise enters through the collective decay ${\gamma }_{12}^{\downarrow }$, which results in different decay rates for the population of the singlet and triplet states, as demonstrated in the upper panel of Fig. 4b, leading to an increase in entanglement. Secondly, the noise enters through the coupling ${{{{\mathcal{J}}}}}_{s}$, which gives rise to oscillation of ${{{\rm{Im}}}}\,{G}_{ts}$, causing the entanglement to also oscillate. This is illustrated in the lower panel of Fig. 4b. To quantitatively analyze how the quantum coherence in the system evolves, we deduce the equation of motion for ${{{{\mathcal{C}}}}}_{R}$ and ${{{{\mathcal{C}}}}}_{I}$, which take the following simple form:

32 $$\begin{array}{l}{\ddot{{{{\mathcal{C}}}}}}_{R}+2{\gamma }^{\downarrow }{\dot{{{{\mathcal{C}}}}}}_{R}+({\gamma }^{\downarrow 2}-{\gamma }_{12}^{\downarrow 2}){{{{\mathcal{C}}}}}_{R}\,=\,0,\\ {\ddot{{{{\mathcal{C}}}}}}_{I}+2{\gamma }^{\downarrow }{\dot{{{{\mathcal{C}}}}}}_{I}+({\gamma }^{\downarrow 2}+4{{{{\mathcal{J}}}}}_{s}^{2}){{{{\mathcal{C}}}}}_{I}\,=\,0,\end{array}$$

representing an overdamped and an underdamped harmonic oscillators, respectively. The local noise *γ*^*↓*^ serves as a “friction” force, impeding the increase of ${{{{\mathcal{C}}}}}_{R}$ and ${{{{\mathcal{C}}}}}_{I}$ and, thus, the entanglement. Surprisingly, the correlated quantum noise (${\gamma }_{12}^{\downarrow }$ and ${{{{\mathcal{J}}}}}_{s}$) acts as an active force that facilitates the increase of entanglement. Considering the initial state $\left\vert \uparrow \downarrow \right\rangle$, these equations can be solved and reveal the following entanglement dynamics encoded in the concurrence:

33 $${{{\mathcal{C}}}}[\rho (t)]={e}^{-{\gamma }^{\downarrow }t}\sqrt{{\sinh }^{2}({\gamma }_{12}^{\downarrow }t)+{\sin }^{2}(2{{{{\mathcal{J}}}}}_{s}t)}.$$

We observe that while the local noise has a detrimental effect on entanglement, the correlated quantum noise-induced correlated decay and coherent coupling both have a beneficial effect. Interestingly, in the absence of coherent coupling ${{{{\mathcal{J}}}}}_{s}=0$, a sizable amount of entanglement can be generated in the pure dissipative evolution due to the correlated decay, corresponding to the blue curve in Fig. 4c. It also provides a lower bound on the entanglement when ${{{{\mathcal{J}}}}}_{s}$ is finite, as evidenced by the green and red curves in Fig. 4c, which display entanglement oscillations with a frequency proportional to ${{{{\mathcal{J}}}}}_{s}$. We remark that, at the long-time evolution limit, the entanglement scales as ${{{\mathcal{C}}}}[\rho (t\to \infty )]\propto \exp [-({\gamma }^{\downarrow }-{\gamma }_{12}^{\downarrow })t]$. This persistence can be attributed to the slow decay of the singlet state, which is long-lived when the correlated noise is comparable to the local noise, ${\gamma }_{12}^{\downarrow }\to {\gamma }^{\downarrow }$.

Let us now study another basic scenario where the DM interaction is present while the symmetric interaction vanishes ${{{{\mathcal{J}}}}}_{s}=0$. The dynamics of the population of the singlet state *G*_*s*_ and of the triplet state *G*_*t*_ are now coupled to each other due to parity-breaking interaction ${{{\mathcal{D}}}}$. The probability flows from the triplet state to singlet state when ${{{\rm{Re}}}}\,{G}_{ts} \,>\, 0$, and in the opposite direction when ${{{\rm{Re}}}}\,{G}_{ts}\,<\, 0$, while the dynamics of ${{{\rm{Re}}}}\,{G}_{ts}$ is governed by the relative population of these states and can be expressed as ${{{\rm{Re}}}}{\partial }_{t}{G}_{ts}=-{\gamma }^{\downarrow }{{{\rm{Re}}}}\,{G}_{ts}+{{{\mathcal{D}}}}({G}_{t}-{G}_{s})$, as shown in Fig. 4d. The element ${{{\rm{Im}}}}\,{G}_{ts}$ is decoupled from other elements and remains zero when the initial state is $\left\vert \uparrow \downarrow \right\rangle$. We solve the coupled dynamics analytically in Supplementary Note 4.

The two-qubit dynamics is affected in two ways by the presence of correlated quantum noise. Firstly, similar to the pure symmetric exchange case, the correlated noise-induced collective decay ${\gamma }_{12}^{\downarrow }$ gives rise to different decay rates of *G*_*t*_ and *G*_*s*_, which can lead to the generation of entanglement. On the other hand, the correlated noise-induced DM interaction ${{{\mathcal{D}}}}$ causes an oscillation between the singlet and triplet state, which can interfere with the effect of ${\gamma }_{12}^{\downarrow }$ in a nontrivial manner. Remarkbaly, one can construct a rather simple equation of motion for ${{{{\mathcal{C}}}}}_{R}$ from the coupled complex dynamics to quantify the entanglement evolution:

34 $${\ddot{{{{\mathcal{C}}}}}}_{R}+2{\gamma }^{\downarrow }{\dot{{{{\mathcal{C}}}}}}_{R}+({\gamma }^{\downarrow 2}-{\gamma }_{12}^{\downarrow 2}+4{{{{\mathcal{D}}}}}^{2}){{{{\mathcal{C}}}}}_{R}=0.$$

**Fig. 5:**
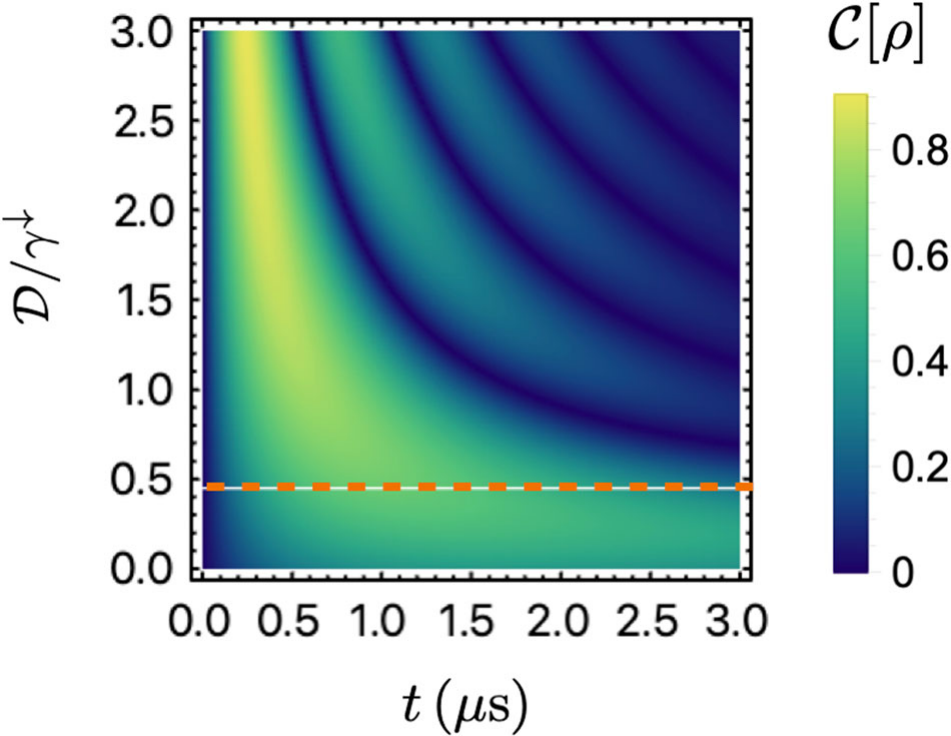
Entanglement dynamics of two driven qubits under DM interaction with initial state $\left\vert \uparrow \downarrow \right\rangle$. The plot shows the time evolution of entanglement as a function of DM interaction strength ${{{\mathcal{D}}}}$. The dashed orange line at ${{{\mathcal{D}}}}=0.45{\gamma }^{\downarrow }$ separates the underdamped and overdamped regimes. When ${{{\mathcal{D}}}}$ is above the dashed line, the entanglement exhibits oscillations with increasing frequency as ${{{\mathcal{D}}}}$ increases. In contrast, below the dashed line, the entanglement decays on a longer timescale and does not exhibit oscillations. Parameters we use in the figure are *γ*^*↓*^ = 1 *μ*s^−1^ and ${\gamma }_{12}^{\downarrow }=0.9{\gamma }^{\downarrow }$.

We observe that the quantum correlated noise-induced collective decay and DM interaction compete with each other, while the local noise *γ*^*↓*^ still acts as a “friction” force as before. When the coherent interaction dominates ${{{\mathcal{D}}}} \,>\, {\gamma }_{12}^{\downarrow }/2$, the system oscillates between the singlet and triplet states, resembling an underdamped oscillator, as illustrated in the upper panel of Fig. 4e. In the regime where ${{{\mathcal{D}}}} \,<\, {\gamma }_{12}^{\downarrow }/2$, the system can be described by an overdamped oscillator. Whenever the probability flows to the triplet state, it quickly decays due to the strong dissipation ${\gamma }^{\downarrow }+{\gamma }_{12}^{\downarrow }$, preventing the probability from returning to the singlet state, and therefore, it does not exhibit any oscillatory behavior, as illustrated in the lower panel of Fig. 4e. With the initial state $\left\vert \uparrow \downarrow \right\rangle$, we solve the entanglement dynamics, which reveals three distinct dynamic regimes:

35 $${{{\mathcal{C}}}}[\rho (t)]={e}^{-{\gamma }^{\downarrow }t}\times \left\{\begin{array}{ll}\left\vert ({\gamma }_{12}^{\downarrow }+2{{{\mathcal{D}}}})\sin {\omega }_{r}t\right\vert /{\omega }_{r}, &2| {{{\mathcal{D}}}}| > {\gamma }_{12}^{\downarrow },\\ 2{\gamma }_{12}^{\downarrow }t, &2| {{{\mathcal{D}}}}| ={\gamma }_{12}^{\downarrow },\\ \left\vert ({\gamma }_{12}^{\downarrow }+2{{{\mathcal{D}}}})\right\vert \sinh \kappa t/\kappa , &2| {{{\mathcal{D}}}}| < {\gamma }_{12}^{\downarrow },\end{array}\right.$$

with ${\omega }_{r}=\sqrt{4{{{{\mathcal{D}}}}}^{2}-{\gamma }_{12}^{\downarrow 2}}$ and $\kappa =\sqrt{{\gamma }_{12}^{\downarrow 2}-4{{{{\mathcal{D}}}}}^{2}}$. In the underdamped regime, the entanglement exhibits oscillations at a frequency of *ω*_*r*_, as illustrated by the green curve in Fig. 4(f), where the odd and even peaks correspond to the singlet and triplet states, respectively. The entanglement decays on a characteristic timescale of 1/*γ*^*↓*^. At the critical point where $2| {{{\mathcal{D}}}}| ={\gamma }_{12}^{\downarrow }$, the oscillation behavior ceases, and the entanglement follows a scaling of $\propto t{e}^{-{\gamma }^{\downarrow }t}$, as depicted by the red curve in Fig. 4f. The loss of oscillation can be clearly observed in Fig. 5, where the dashed orange line represents the critical point; entanglement exhibits oscillations above this point, while there is no oscillation below it. In the overdamped regime, the entanglement exhibits a scaling behavior of $\propto \exp [-({\gamma }^{\downarrow }-\kappa )t]$, with an extended lifetime of 1/(*γ*^*↓*^ − *κ*), while the maximal entanglement it can reach is reduced in the dynamics, as shown by the blue curve in Fig. 4f.

Our findings indicate that while noise typically poses challenges in quantum information processing, correlated quantum noise, when effectively utilized, can become a key resource for generating substantial long-lived entanglement. Importantly, the entanglement generation process can be precisely controlled on-demand by simply switching the driving on and off.

Contrasting with idle qubits that lack driving and cannot develop coherence through correlated dephasing dynamics, we interestingly observe that correlated decay, induced by transverse noise, can generate substantial entanglement. This occurs as the decay rates of singlet and triplet states differ. Notably, while both arise from correlated quantum noise and contribute to entanglement generation, the interplay between correlated decay and coherent coupling results in distinct dynamical regimes. From the two fundamental cases presented here, one can extrapolate to dynamics when both symmetric exchange and DM interaction are present, and we leave the detailed discussion of this situation to Supplementary Note 4.

### Driven spin qubits subject to correlated 1/*f* noise

Building upon the insights obtained from the previous study on Markovian noise, in this section, we investigate the impact of spatially and temporally correlated 1/*f* noise, which is non-Markovian in nature. We assume that the two driven qubits are located within a few hundred nanometers of each other, and thus the spatially correlated noise is comparable to the local noise *S*_12_ ≈ *S*_*ii*_ in the relevant frequency range. We present an analytical investigation into two situations, in the first one, only classical 1/*f* noise is present, while in the second one, we include also quantum 1/*f* noise, comparable to the classical noise. This situation can occur at low temperatures, when *k*_*B*_*T*≤*ℏ*Ω, where the ability of qubits to emit and absorb energy is different. For simplicity, we assume that the spectral density of the correlated noise is real, and we focus on its temporal correlations. The detailed derivations of the results in this section are sketched in Supplementary Note 5.

In the presence of purely classical 1/*f* noise, the two-qubit dynamics is governed by the TCL master equation (17) with vanishing coherent coupling ${{{\mathcal{J}}}}(t)=0$ and equal local and correlated absorption and decay rates, denoted as $\gamma (t)\equiv {\gamma }_{ij}^{\downarrow }={\gamma }_{ij}^{\uparrow }$, with following form:

36 $$\begin{array}{ll}\gamma (t)\,=\,\frac{2}{{\hslash }^{2}}\int\nolimits_{0}^{\infty }\frac{d\omega }{2\pi }{S}_{ii}^{C}(\omega )\left[{F}_{s}(\omega -\Omega ,t)+{F}_{s}(\omega +\Omega ,t)\right]\\\quad\quad\,=\,\frac{4{\sigma }^{2}}{{\hslash }^{2}\Omega }\left[{{{\rm{Si}}}}(\Omega t)-\sin (\Omega t){{{\rm{Ci}}}}({\omega }_{l}t)\right].\end{array}$$

**Fig. 6:**
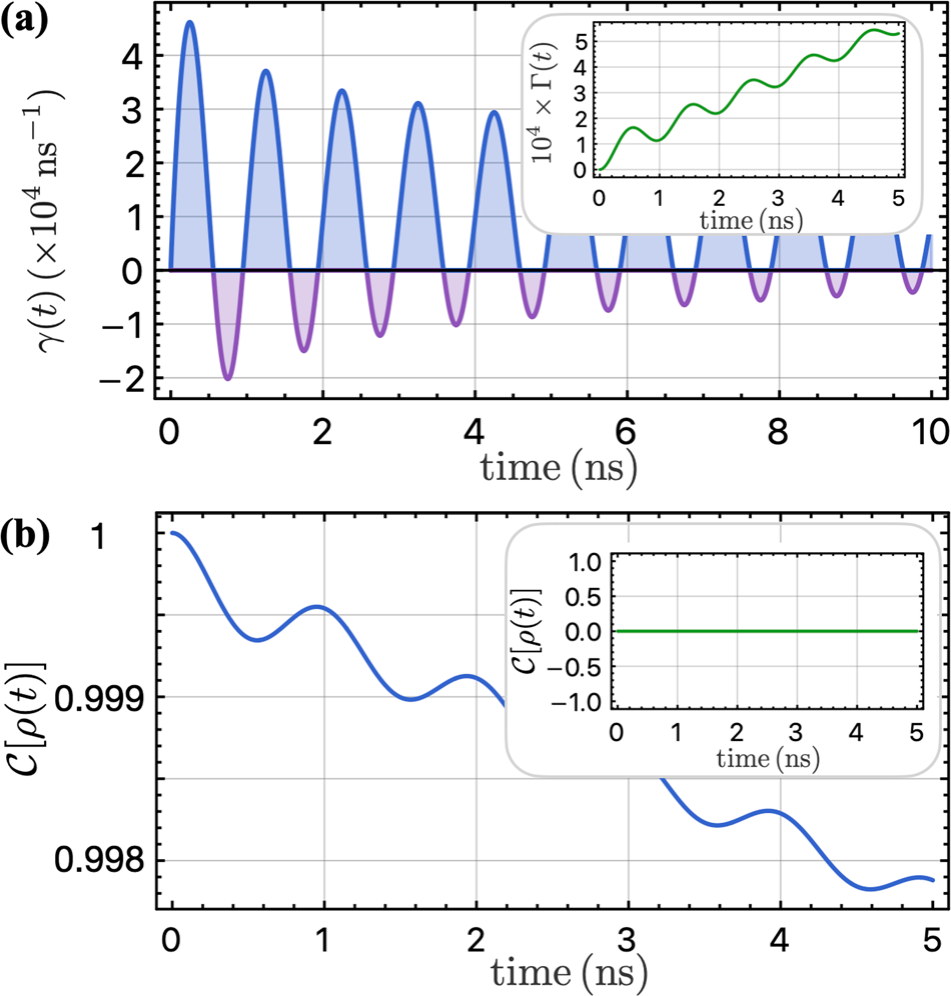
Entanglement dynamics of two driven qubits under pure classical 1/*f* noise. **a** Temporal evolution of the decay rate *γ*(*t*) depicted as a function of time, where the occurrence of negative values is identified within the marked purple intervals. However, the integral of the decay rate Γ(*t*), as highlighted in the inset of **a**, must remain nonnegative to ensure the complete positivity of the dynamics. **b** Entanglement between two qubits as a function of time with the initial state being a Bell state $\left\vert {\psi }_{0}\right\rangle =(\left\vert \uparrow \downarrow \right\rangle +i\left\vert \downarrow \uparrow \right\rangle )/\sqrt{2}$, and the inset shows the entanglement with initial state $\left\vert \uparrow \downarrow \right\rangle$. Parameters that are used in the plots: *ℏ*/*σ* = 100 ns, *ω*_*l*_/2*π* = 1 MHz, and Ω/2*π* = 1 GHz.

Here, ${{{\rm{Si}}}}(x)\equiv \int\nolimits_{0}^{x}d\tau \sin (\tau )/\tau$ is the sine integral function. One surprising feature of the rate *γ*(*t*) is that it can take negative values during finite time intervals, denoted by the purple regions in Fig. 6a. This behavior is an indication of non-Markovian memory effects, reflecting the exchange of information between the two qubits and the environment^67^. Nevertheless, the time integral of *γ*(*t*), denoted as

37 $$\Gamma (t)\equiv \int\nolimits_{0}^{t}ds\,\gamma (s),$$

must remain non-negative due to the complete positivity requirement of the system dynamics^52,93^. This is illustrated in the inset of Fig. 6a.

Based on the insights gained from the previous sections, we anticipate that entanglement generation would not be possible with purely classical correlated noise. This is indeed the case even when the noise is temporally correlated, as shown in the inset of Fig. 6b, where the entanglement remains to be zero with a trivial initial product state $\left\vert \uparrow \downarrow \right\rangle$. A new feature arising from the non-Markovian nature is the occurrence of oscillations in the decay of entanglement, when the initial state is entangled. For concreteness, we initialize the system to be the Bell state $\left\vert {\psi }_{0}\right\rangle =(\left\vert \uparrow \downarrow \right\rangle +i\left\vert \downarrow \uparrow \right\rangle )/\sqrt{2}$. The dynamics of the entanglement is then given by

38 $${{{\mathcal{C}}}}[\rho (t)]=\frac{1}{3}\left[\sqrt{4{e}^{-6\Gamma (t)}{\sinh }^{2}(3\Gamma )+9{e}^{-4\Gamma (t)}}+{e}^{-6\Gamma (t)}-1\right],$$

which is depicted in Fig. 6b. To gain some insights, let us consider a specific quantum trajectory. During time intervals where the rate *γ*(*t*) > 0, the system can undergo quantum jumps, which can lead to the loss of coherence and a transition from an entangled state such as $\left\vert {\psi }_{0}\right\rangle$ to a trivial state like $\left\vert \downarrow \downarrow \right\rangle$ with a finite probability. Conversely, during the negative rate *γ*(*t*) < 0 later on, the quantum jump process can be interpreted as a jump in the reverse direction^67,94–96^, $\left\vert {\psi }_{0}\right\rangle \leftarrow \left\vert \downarrow \downarrow \right\rangle$, with a finite probability of restoring the lost superposition due to the non-Markovian memory effect. As a result, the entanglement exhibits a temporary increase during the decoherence process, as illustrated in Fig. 6b.

We now investigate the impact of quantum correlated noise in the quantum regime where *S*^*Q*^ ≈ *S*^*C*^. The dynamics of the two-qubit system is governed by the same master equation (17), with the decay and absorption rates defined as

39 $$\begin{array}{ll}{\gamma }_{ij}^{\downarrow }(t)\,=\,\frac{4}{{\hslash }^{2}}\int\nolimits_{0}^{\infty }\frac{d\omega }{2\pi }{S}_{ij}^{Q}(\omega ){F}_{s}(\omega -\Omega ,t),\\ {\gamma }_{ij}^{\uparrow }(t)\,=\,\frac{4}{{\hslash }^{2}}\int\nolimits_{0}^{\infty }\frac{d\omega }{2\pi }{S}_{ij}^{Q}(\omega ){F}_{s}(\omega +\Omega ,t).\end{array}$$

Considering that the filter function *F*_*s*_(*ω* + Ω, *t*) is peaked at *ω* = − Ω which lies outside the range of integration, we approximate the absorption rate ${\gamma }_{ij}^{\uparrow }$ to be zero to enable complete analytical solution of the dynamics. In the strong correlated noise regime considered in this section, we assume that the local and correlated decay rates are equal and we denote them as ${\gamma }^{\downarrow }(t)\equiv {\gamma }_{ij}^{\downarrow }(t)$, which is evaluated to be:

40 $${\gamma }^{\downarrow }(t)=\frac{4{\sigma }^{2}}{{\hslash }^{2}\Omega }\left[\pi (1-\cos \Omega t)/2-\sin \Omega t{{{\rm{Ci}}}}({\omega }_{l}t)+{{{\rm{Si}}}}(\Omega t)\right].$$

**Fig. 7:**
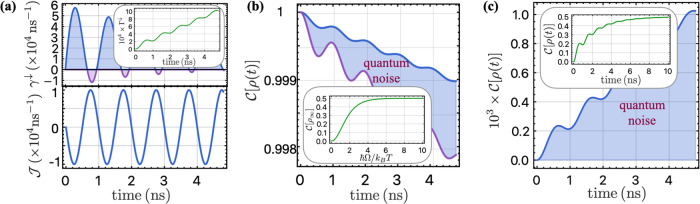
Analysis of driven qubit dynamics in the presence of quantum noise. **a** The upper panel depicts the time-dependent decay rate *γ*^*↓*^, which can take negative values for certain time intervals (indicated by purple shading). Its time integral Γ^*↓*^ is positive at all times to ensure the complete positivity of the dynamics. The lower panel shows the coherent coupling ${{{\mathcal{J}}}}$ between two qubits as a function of time. **b** Time-dependent entanglement for a maximally entangled initial state $\left\vert {\psi }_{0}\right\rangle =(\left\vert \uparrow \downarrow \right\rangle +i\left\vert \downarrow \uparrow \right\rangle )/\sqrt{2}$. The blue shaded area demonstrates the impact of quantum noise, with the blue and purple curves representing cases with and without quantum noise, respectively. The inset displays the final steady-state entanglement as a function of temperature, which is zero in the classical limit and 1/2 in the quantum regime. **c** Entanglement evolution as a function of time for an initial state $\left\vert \uparrow \downarrow \right\rangle$. The effect of quantum noise is illustrated by the blue shaded region (the entanglement remains at zero when only classical noise is present). The inset reveals a final entanglement value of 1/2 with *ℏ*/*σ* = 3 ns within a time ~ 10 ns. The plotted results are obtained using the following parameters: *ℏ*/*σ* = 100 ns, *ω*_*l*_/2*π* = 1 MHz, and Ω/2*π* = 1 GHz.

The upper panel of Fig. 7a shows that the rate *γ*^*↓*^(*t*) can be negative temporarily, while its time integral defined as

41 $${\Gamma }^{\downarrow }(t)\equiv \int\nolimits_{0}^{t}ds\,{\gamma }^{\downarrow }(s),$$

must be positive due to the complete positivity of the dynamics, as illustrated in the inset. In the presence of quantum correlated noise, the coherent coupling ${{{\mathcal{J}}}}(t)$ in the Hamiltonian (18) is nonvanishing and given by:

42 $$\begin{array}{l}{{{\mathcal{J}}}}(t)\,=\,\frac{2}{{\hslash }^{2}}\int\nolimits_{0}^{\infty }\frac{d\omega }{2\pi }{S}^{Q}(\omega )\left[{F}_{c}(\omega -\Omega ,t)+{F}_{c}(\omega +\Omega ,t)\right]\\\quad\quad\,\,=\,-\frac{2\pi {\sigma }^{2}}{{\hslash }^{2}\Omega }\sin \Omega t,\end{array}$$

which oscillates with a frequency of Ω and takes values comparable to the decay rate as shown in the lower panel of Fig. 7a. To demonstrate the effect of quantum noise, we first initialize the system into the Bell state $\left\vert {\psi }_{0}\right\rangle =(\left\vert \uparrow \downarrow \right\rangle +i\left\vert \downarrow \uparrow \right\rangle )/\sqrt{2}$ and investigate how the entanglement decays in the presence of both classical and quantum 1/*f* noise. We show the entanglement in this case is given by

43 $${{{\mathcal{C}}}}[\rho (t)]={e}^{-{\Gamma }^{\downarrow }(t)}\sqrt{{\sinh }^{2}{\Gamma }^{\downarrow }(t)+{\cos }^{2}\Phi (t)},$$

with the phase Φ(*t*) defined as $\Phi (t)\equiv \int\nolimits_{0}^{t}ds\,{{{\mathcal{J}}}}(s)=2\pi {\sigma }^{2}(\cos \Omega t-1)/{\hslash }^{2}{\Omega }^{2}.$ It is shown as the blue curve in Fig. 7b, with the purely classical 1/*f* noise case also shown for contrast as the purple curve. The temporary increase in the entanglement during the decoherence process is also observed in the presence of quantum correlated noise. In the case of strong correlated quantum 1/*f* noise, decoherence occurs at a much slower rate, and the net effect of quantum noise is reflected in the shaded blue region in Fig. 7b. The entanglement is long-lived, and the final entanglement approaches 1/2 due to the long-lived singlet state with a decay rate of ${\gamma }_{ii}^{\downarrow }-{\gamma }_{12}^{\downarrow }$, which is almost zero when the correlated noise is comparable to the local noise. For arbitrary temperature, the residual entanglement is shown to be:

44 $$\begin{array}{l}{{{\mathcal{C}}}}[{\rho }_{\infty }]\,=\,\frac{1}{2}-\frac{3}{4\cosh (\beta \hslash \Omega )+2}\\\qquad\quad=\,\frac{2{[{S}^{Q}(\Omega )]}^{2}}{3{[{S}^{C}(\Omega )]}^{2}+{[{S}^{Q}(\Omega )]}^{2}},\end{array}$$

which is shown in the inset of Fig. 7b. Here we have invoked the fluctuation-dissipation theorem ${S}_{ij}^{C}(\omega )=\coth (\beta \hslash \omega /2){S}_{ij}^{Q}(\omega )$. It can be clearly observed that in the classical limit where *k*_*B*_*T* ≫ *ℏ*Ω, *S*^*Q*^ = 0, the final entanglement is zero as expected. Furthermore, we also conclude that there is always a finite, long-lasting entanglement present when the correlated quantum noise (comparable to local quantum noise) is finite.

To investigate the entanglement generation by spatially correlated quantum 1/*f* noise, we consider a simple initial state $\left\vert \uparrow \downarrow \right\rangle$. The entanglement then is given by

45 $${{{\mathcal{C}}}}[\rho (t)]={e}^{-{\Gamma }^{\downarrow }(t)}\sqrt{{\sinh }^{2}{\Gamma }^{\downarrow }(t)+{\sin }^{2}\Phi (t)}.$$

We note that there are temporary decrease in the growing entanglement, which finally reaches 1/2, as shown in Fig. 7c and its inset. This can also be attributed to the non-Markovian memory effect. Considering a quantum trajectory, when the decay rate is positive *γ*^*↓*^(*t*) > 0, the system can undergo quantum jumps, taking the product state to an entangled state in the presence of correlated quantum noise. Later, when the decay rate is negative *γ*^*↓*^(*t*) < 0, the state jumps back to a product state from the entangled state, which leads to the temporary dip in the entanglement growth. Our findings demonstrate the potential of utilizing the correlated quantum 1/*f* noise, which is ubiquitous in solid-state quantum computing platforms, to generate significant entanglement or delay the decoherence process in two-qubit systems. We also illustrate the effects of the non-Markovianity of the noise in the dynamics of two driven qubits.

### Comparison with the Single Qubit Case

After investigating the entanglement dynamics between two qubits in the presence of the correlated noise, it is helpful to compare it with the coherence decay in a single qubit case. We first consider an idle qubit without driving. In this case, its dynamics is governed by the following master equation in the interaction picture, according to Eq. (12),

46 $$\dot{\rho }(t)={\gamma }^{z}(t)[{\sigma }^{z}\rho {\sigma }^{z}-\rho ],$$

where *ρ* is the density matrix for the qubit, and the local dephasing rate experienced by the qubit is

47 $$\begin{array}{l}{\gamma }^{z}(t)\,=\,\frac{4}{{\hslash }^{2}}\int\nolimits_{0}^{\infty }\frac{d\omega }{2\pi }\frac{\sin (\omega t)}{\omega }\frac{2\pi {\sigma }^{2}}{\omega }\\\qquad\quad=\,\frac{4{\sigma }^{2}t}{{\hslash }^{2}}\left[1-{{{\rm{Ci}}}}({\omega }_{l}t)\right].\end{array}$$

The diagonal elements of *ρ* do not evolve in the pure dephasing noise. The off-diagonal element, representing the superposition of two states of the qubit, decays as

48 $$\begin{array}{ll}{\rho }_{12}(t)\,=\,{\rho }_{12}(0)\exp \left(-2\int\nolimits_{0}^{t}ds\,{\gamma }^{z}(s)\right)\\\qquad\quad=\,{\rho }_{12}(0)\exp \left[-\frac{2{\sigma }^{2}{t}^{2}}{{\hslash }^{2}}\left(3-2\gamma -2\ln ({\omega }_{l}t)\right)\right],\end{array}$$

which is the well-known Gaussian decay with a logarithmic correction. We note that the presence of correlated noise in the two-qubit case [as detailed in the discussion above Eq. (25)] only modifies the prefactor of the decay function of the off-diagonal elements. We point out that, while the local noise spectral density corresponds to the local correlator of the environment and the correlated noise spectral density is linked to the nonlocal correlator in real space, both types of spectral density encapsulate information about the correlation of the environment. This is evident from Eq. (9), where *S*_*ij*_(*ω*) includes contributions from excitations in the environment possessing finite wavelengths.

When the qubit is resonantly driven, its dynamics is described by the master equation:

49 $$\begin{array}{ll}\dot{\rho }\,=\,{\gamma }^{\downarrow }(t)\left[{\hat{\sigma }}^{-}\rho {\hat{\sigma }}^{+}-\frac{1}{2}\{{\hat{\sigma }}^{+}{\hat{\sigma }}^{-},\rho \}\right]\\\quad\quad+\,{\gamma }^{\uparrow }(t)\left[{\hat{\sigma }}^{+}\rho {\hat{\sigma }}^{-}-\frac{1}{2}\{{\hat{\sigma }}^{-}{\hat{\sigma }}^{+},\rho \}\right],\end{array}$$

in the interaction picture according to Eq. (17). We consider the scenario where both quantum and classical noise are present, *S*^*Q*^(*ω*) ≈ *S*^*C*^(*ω*) = 2*πσ*^2^/*ω*. Then the decay and absorption rates are

50 $$\begin{array}{ll}{\gamma }^{\downarrow }(t)\,=\,\frac{4}{{\hslash }^{2}}\int\nolimits_{0}^{\infty }\frac{d\omega }{2\pi }\frac{2\pi {\sigma }^{2}}{\omega }{F}_{s}(\omega -\Omega ,t)\\ {\gamma }^{\uparrow }(t)\,=\,\frac{4}{{\hslash }^{2}}\int\nolimits_{0}^{\infty }\frac{d\omega }{2\pi }\frac{2\pi {\sigma }^{2}}{\omega }{F}_{s}(\omega +\Omega ,t).\end{array}$$

Similar to the approximation that we made in Eq. (39), we approximate the absorption rate to be zero since *F*_*s*_(*ω* + Ω, *t*) is sharply peaked at *ω* = − Ω which is outside the integration range. The explicit expression of the decay rate is given in Eq. (40). The off-diagonal element decays as:

51 $${\rho }_{12}(t)={\rho }_{12}(0)\exp \left(-\frac{1}{2}\int\nolimits_{0}^{t}ds\,{\gamma }^{\downarrow }(s)\right).$$

We can also write down the evolution of $\langle {\hat{\sigma }}_{x}\rangle$ (in Heisenberg picture):

52 $$\begin{array}{ll}\langle {\hat{\sigma }}_{x}\rangle (t)\,=\,\left[\langle {\sigma }^{x}\rangle (0)\cos (\Omega t)-\langle {\sigma }^{y}\rangle (0)\sin (\Omega t)\right]\\\qquad\qquad\times \exp \left(-\frac{1}{2}\int\nolimits_{0}^{t}ds\,{\gamma }^{\downarrow }(s)\right).\end{array}$$

Compared with the dynamics observed in the two-qubit scenario, it is notable that the correlated noise dramatically alters the dynamics, distinctly different from its impact on idle qubits. In particular, the correlated noise introduces an asymmetry in the decay rates of the singlet and triplet states. This asymmetry results in the exponential growth factor observed in the entanglement dynamics in Eqs. (33), (35), (43), and (45), as elaborated in the previous sections.

## Discussion

In this paper, we have presented a comprehensive analytical study of the two-qubit dynamics subject to both local and non-local spatially correlated noise. Our analysis is based on a time-local TCL master equation that is applicable for generic noise spectra, including both Markovian and non-Markovian noise. We explored how the classical and quantum correlations stored in the noise dictate the qubit dynamics. Our study shows that at temperatures higher than the Rabi frequency of the qubit, where classical noise dominates, correlated noise affects only the decoherence rate without causing qubit entanglement. This suggests that higher operating temperatures can effectively reduce crosstalk between qubits due to correlated noise^66^.

In the case of low temperatures, when both classical and quantum correlations are present in the noise, the correlated quantum noise introduces various new effects. These include the coherent Ising interaction and correlated dephasing in the case of purely dephasing noise, as well as the coherent symmetric exchange, DM interaction, and correlated relaxation in the case of transverse noise. We have illustrated the effects of these interaction by solving the two-qubit dynamics analytically. Specifically, our analysis has demonstrated that only the noise-induced Ising interaction, not the correlated dephasing, can lead to entanglement with finite lifetime. In the case of transverse noise, our findings indicate that coherent interactions and correlated relaxation not only generate substantial entanglement but also notably extend its lifetime. Moreover, this process can be on-demand activated or deactivated through coherent qubit drive. We finally studied the non-Markovian memory effect by investigating the correlated 1/*f* noise, where the decay rate can be negative temporarily.

Our research underscores the advantageous potential of operating qubits at higher temperatures in scenarios involving correlated noise. Additionally, we emphasize the significance of employing resonant qubit drive at lower temperatures, which acts as an effective switch for turning on and off the generation of long-lived entanglement. This approach leverages correlated noise as a valuable resource.

Future work will explore the effect of long-ranged noise, beyond nearest-neighbor, in multiple qubit systems. This case is critical for future experiments aiming to scale up quantum processors, especially because recent measurements highlighted the presence of long-range noise in spin qubits in quantum dots^62^. Investigating how the correlated noise affects standard quantum operations, such as the fidelity of two-qubit gates is therefore critical^52^. Additionally, it is important to understand how the correlated noise interferes with existing strategies developed to suppress the impact of single-qubit noise, such as quantum error correction codes, dynamical decoupling, and sweet spots. Our work provides a solid foundation for future research in these directions and will serve as a starting point to address these challenges towards large-scale quantum computers.

## Methods

### Analytical formalism

Our analysis relies on the time-convolutionless projection operator approach. Let us consider a system of our interest coupled to an environment that we will not keep track of. The dynamics of the combined system is governed by a microscopic Hamiltonian of the form:

53 $$H={H}_{{{{\rm{S}}}}}+{H}_{{{{\rm{E}}}}}+{H}_{{{{\rm{SE}}}}},$$

where *H*_S_ and *H*_E_ dictate the time evolution of the system and the environment, respectively, and *H*_SE_ describes the coupling between them. In the platform considered in the main text, *H*_S_ is the two-qubit system that is of interest and *H*_E_ is the environment that gives rise to local and correlated noise, leading to decoherence of qubits via the coupling *H*_SE_. It is convenient to work in the interaction picture, where the density matrix of the combined system *ρ*_tot_(*t*) obeys the following equation of motion:

54 $$\frac{{{{\rm{d}}}}{\rho }_{{{{\rm{tot}}}}}(t)}{{{{\rm{d}}}}t}={{{\mathcal{L}}}}(t){\rho }_{{{{\rm{tot}}}}}(t),$$

with the Liouville superoperator defined as ${{{\mathcal{L}}}}(t){\rho }_{{{{\rm{tot}}}}}(t)\equiv -i[{H}_{{{{\rm{SE}}}}}(t),{\rho }_{{{{\rm{tot}}}}}(t)]/\hslash$ and the coupling in interaction picture ${H}_{{{{\rm{SE}}}}}(t)\equiv \exp [i({H}_{{{{\rm{S}}}}}+{H}_{{{{\rm{E}}}}})t/\hslash ]{H}_{{{{\rm{SE}}}}}\exp [-i({H}_{{{{\rm{S}}}}}+{H}_{{{{\rm{E}}}}})t/\hslash ]$. We aim to derive the equation of motion for the reduced density matrix *ρ* of the system. To this end, we introduce a projection superoperator ${{{\mathcal{P}}}}$ that projects any density matrix *ρ*_tot_ onto the system part of the Hilbert space: ${{{\mathcal{P}}}}{\rho }_{{{{\rm{tot}}}}}\equiv {{{{\rm{tr}}}}}_{{{{\rm{E}}}}}[{\rho }_{{{{\rm{tot}}}}}]\otimes {\rho }_{{{{\rm{B}}}}}$, where the trace is taken over the environment and *ρ*_B_ is the initial state of the environment which we take to be the thermal state in the main text. We note that our goal is to obtain a closed equation for ${{{\mathcal{P}}}}{\rho }_{{{{\rm{tot}}}}}$, which would give us the equation for *ρ* naturally. Accordingly, a complementary superoperator ${{{\mathcal{Q}}}}$ can be defined by ${{{\mathcal{Q}}}}\equiv {{{\mathcal{I}}}}-{{{\mathcal{P}}}}$, with the identity operator ${{{\mathcal{I}}}}$, which projects on the irrelevant part of the density matrix. By applying the projection operators ${{{\mathcal{P}}}}$ and ${{{\mathcal{Q}}}}$ to the Liouville-von Neumann equation (54), we arrive at

55 $$\begin{array}{ll}\frac{{{{\rm{d}}}}}{{{{\rm{d}}}}t}{{{\mathcal{P}}}}{\rho }_{{{{\rm{tot}}}}}(t)\,=\,{{{\mathcal{P}}}}{{{\mathcal{L}}}}(t){{{\mathcal{P}}}}{\rho }_{{{{\rm{tot}}}}}(t)+{{{\mathcal{P}}}}{{{\mathcal{L}}}}(t){{{\mathcal{Q}}}}{\rho }_{{{{\rm{tot}}}}}(t),\\ \frac{{{{\rm{d}}}}}{{{{\rm{d}}}}t}{{{\mathcal{Q}}}}{\rho }_{{{{\rm{tot}}}}}(t)\,=\,{{{\mathcal{Q}}}}{{{\mathcal{L}}}}(t){{{\mathcal{P}}}}{\rho }_{{{{\rm{tot}}}}}(t)+{{{\mathcal{Q}}}}{{{\mathcal{L}}}}(t){{{\mathcal{Q}}}}{\rho }_{{{{\rm{tot}}}}}(t).\end{array}$$

The idea now is to solve for ${{{\mathcal{Q}}}}{\rho }_{{{{\rm{tot}}}}}(t)$ formally and substitute it into the equation for ${{{\mathcal{P}}}}{\rho }_{{{{\rm{tot}}}}}$, which results in a closed equation for ${{{\mathcal{P}}}}{\rho }_{{{{\rm{tot}}}}}$. At this point, there are typically two ways to proceed, both of which give rise to exact but conceptually different equations of motion for the reduced density matrix. These methods are detailed in Ref. ^88^. One approach leads to the well-known Nakajima-Zwanzig equation, which is a time-non-local equation containing a memory kernel. In contrast, the second method yields a time-convolutionless master equation with the following form:

56 $$\frac{{{{\rm{d}}}}}{{{{\rm{d}}}}t}{{{\mathcal{P}}}}{\rho }_{{{{\rm{tot}}}}}={{{\mathcal{K}}}}(t){{{\mathcal{P}}}}{\rho }_{{{{\rm{tot}}}}}(t),$$

where ${{{\mathcal{K}}}}(t)$ is a time-local generator, known as the TCL generator, and we have assumed the initial state of the combined system takes the form of *ρ*_tot_(0) = *ρ*(0) ⊗ *ρ*_B_ thus ${{{\mathcal{Q}}}}{\rho }_{{{{\rm{tot}}}}}(0)=0$. As customary, we assume ${{{{\rm{tr}}}}}_{{{{\rm{E}}}}}[{H}_{{{{\rm{SE}}}}}(t){\rho }_{{{{\rm{B}}}}}]=0$ (namely, the noise operator *E*_*i*_ in the main text has vanishing mean in state *ρ*_B_) and also assume that coupling between the system and the environment is sufficiently weak. Thus we can truncate the TCL generator ${{{\mathcal{K}}}}(t)$ at second order, yielding:

57 $${{{\mathcal{K}}}}(t)=\int\nolimits_{0}^{t}ds\,{{{\mathcal{P}}}}{{{\mathcal{L}}}}(t){{{\mathcal{L}}}}(s){{{\mathcal{P}}}}.$$

Therefore, the TCL master equation for the reduced density matrix *ρ* in the interaction picture takes the form of

58 $$\frac{{{{\rm{d}}}}}{{{{\rm{d}}}}t}\rho (t)=\int\nolimits_{0}^{t}ds\,{{{{\rm{tr}}}}}_{{{{\rm{E}}}}}\left[{{{\mathcal{L}}}}(t){{{\mathcal{L}}}}(s)\rho (t)\otimes {\rho }_{{{{\rm{B}}}}}\right].$$

This equation serves as the starting point of our analysis.

### Supplementary information

Supplementary Information

## Data Availability

All data supporting the findings of this study are obtained from analytical solutions, which are available within the article and its Supplementary Information.

## Code availability

All figures in this study are generated using known analytical expressions. The detailed code is available from the corresponding author upon request.
