# Supplementary material for: Spatially correlated classical and quantum noise in driven qubits
Source: npj Quantum Inf. 2024 Apr 30;10(1):46. doi: 10.1038/s41534-024-00842-9 (PMC11062932; doi:10.1038/s41534-024-00842-9)
Supplement: Supplementary file 1 — Supplementary Information [file 41534_2024_842_MOESM1_ESM.pdf]

Supplementary Information for
" Spatially correlated classical and quantum noise in driven qubits"

Ji Zou,[1,*] Stefano Bosco,[1] and Daniel Loss[1]

[1]Department of Physics, University of Basel, Klingelbergstrasse 82, 4056 Basel, Switzerland

SUPPLEMENTARY NOTE 1: SPATIALLY CORRELATED NOISE IN DIFFERENT DIMENSIONS

In the main text, we introduce the local and spatially correlated noise spectral densities $S_{ij}(\omega)$. To be specific, we present the relation between the local and correlated noise in two dimensional architectures in the main text. In this section, we provide a detailed illustration of their relations in various dimensions, assuming a linear spectrum of the environment as $\omega_{\mathbf{k}} = c_s|\mathbf{k}|$. This allows us to write the noise spectral density as:

$$\begin{aligned} S_{ij}(\omega) =& 2\pi|g_k|^2[n_B(\omega)+1]\sum_{\mathbf{k}} e^{-i\mathbf{k}\cdot(\mathbf{r}_j-\mathbf{r}_i)}\delta(\omega-\omega_k) \\ &+ 2\pi|g_{-k}|^2 n_B(-\omega)\sum_{\mathbf{k}} e^{i\mathbf{k}\cdot(\mathbf{r}_j-\mathbf{r}_i)}\delta(\omega+\omega_k), \end{aligned} \tag{1}$$

where $k=\omega/c_s$ and we have assumed the coupling $g_{\mathbf{k}}$ only depends on the magnitude of $\mathbf{k}$. The first term (positive-frequency component) represents the emission of energy into the environment, while the second term (negative-frequency component) stands for the absorption of energy from the environment. It is evident that the summation over the momentum $\mathbf{k}$ yields distinct outcomes in various dimensions. After some algebraic manipulation, we obtain the following conclusions:

$$\begin{aligned} &\text{1D}: \;\; S_{12}(\omega) = \cos(kd)S_{ii}(\omega), \\ &\text{2D}: \;\; S_{12}(\omega) = J_0(kd)S_{ii}(\omega), \\ &\text{3D}: \;\; S_{12}(\omega) = \frac{\sin(kd)}{kd}S_{ii}(\omega). \end{aligned} \tag{2}$$

Here, $J_0(kd)$ represents the Bessel function of the first kind, which decays algebraically at large distances as $J_0(kd) \propto \cos(kd-\pi/4)/\sqrt{kd}$. From this, it is evident that the correlated noise is always bounded by the local noise, that is, $|S_{12}| \leq S_{ii}$. Finally, we remark that we have assumed that the quasiparticle does not decay when traveling between the two spins.

SUPPLEMENTARY NOTE 2: TIME CONVOLUTIONLESS MASTER EQUATIONS

In the section of Results of the main text, we have presented the TCL master equations for the two-qubit system, which allow us to investigate the effects of correlated classical and quantum noise. In this section, we provide detailed derivations for the results presented in the main text. Based on the time-convolutionless projection operator method presented in the Methods section, we derive the master equation for pure-dephasing noise and the master equation for pure-transverse noise.

Master equation for pure-dephasing noise. Here, we drive the TCL master equations for the two-qubit system in the absence of the coherent drive. In this scenario, the qubit-environment coupling is given by

$$H_{\rm SE} = \sum_{i\in\{1,2\}} \sigma_i^z E_i, \tag{3}$$

where E_i are the noise operators acting on the Hilbert space of the environment. With this interaction, we can derive the master equation by utilizing the TCL equation:

$$\begin{aligned} \frac{\mathrm{d}\rho(t)}{\mathrm{d}t} =& -\frac{1}{\hbar^2}\sum_{i,j}\int_0^t ds\,\big[S_{ij}(t-s)(\sigma_i^z\sigma_j^z\rho - \sigma_j^z\rho\sigma_i^z) \\ &+ S_{ij}(s-t)(\rho\sigma_i^z\sigma_j^z - \sigma_j^z\rho\sigma_i^z)\big], \end{aligned} \tag{4}$$

where we have introduced the two-point correlation function of the noise operators $\mathrm{tr}_E[E_i(t)E_j(s)\rho_B] = \mathrm{tr}_E[E_i(t-s)E_j\rho_B] \equiv S_{ij}(t-s)$. Here we have used the fact that the initial state of the environment is the thermal state which is stationary. We note that terms with $i = j$ correspond to the standard local dephasing dynamics rooted in the local noise, whereas the terms with $i \neq j$ describe the collective dynamics originated from the correlated noise. We can cast the equation above into the following form:

$$\begin{aligned}\frac{\mathrm{d}\rho(t)}{\mathrm{d}t} = &-\frac{1}{2\hbar^2}\sum_{i,j}\int_0^t ds\,\big[S_{ij}(s) - S_{ij}(-s)\big][\sigma_i^z\sigma_j^z, \rho] \\ &+ \frac{1}{\hbar^2}\int_0^t ds\,\big[S_{ij}(s) + S_{ij}(-s)\big]\mathcal{D}_{ij}^z[\rho],\end{aligned} \tag{5}$$

where the dissipator is defined as usual: $\mathcal{D}_{ij}^z[\rho] \equiv \sigma_j^z\rho\sigma_i^z - \{\sigma_i^z\sigma_j^z, \rho\}/2$. We remark that there is also a term proportional to σ_i^z, which describes the induced Lamb shift and renormalizes the qubit energy spliting. It is rooted in the local noise and we thus have neglected it since our main concern is the correlated noise. Then we can conveniently write the master equation above into the following form:

$$\dot{\rho}(t) = -i[H_z(t), \rho(t)] + \sum_{i,j\in\{1,2\}}\mathcal{L}_{ij}^z(t)\rho(t). \tag{6}$$

Below, we focus on the coherent part (which leads to unitary evolution) and the dissipative part (which gives rise to non-unitary dynamics), respectively.

Coherent interaction. The environment-induced coherent Ising interaction takes the form of $H_z(t) = \mathcal{J}^z(t)\sigma_1^z\sigma_2^z$ with the coupling being

$$\begin{aligned}\mathcal{J}^z(t) &= \frac{1}{i\hbar^2}\sum_{i\neq j}\int_0^t ds\,\frac{S_{ij}(s) - S_{ij}(-s)}{2} \\ &= \frac{1}{2\hbar^2}\int_0^t ds\,\big[\mathcal{G}_{12}^R(s) + \mathcal{G}_{21}^R(s)\big],\end{aligned} \tag{7}$$

where $\mathcal{G}_{12}^R(s) \equiv -i\Theta(s)\langle[E_1(s), E_2]\rangle$ represents the standard retarded Green's function, which indicates the retarded interaction between the two qubits mediated by the environment. One can easily check that this coupling function is real-valued according to its definition above. We aim to examine the effect of the correlated quantum and classical noise. It is helpful to write the expression in a more suggestive form by utilizing $S_{ij}(t) = \int d\omega\, e^{-i\omega t}S_{ij}(\omega)/2\pi$,

$$\mathcal{J}(t) = \frac{1}{\hbar^2}\int_{-\infty}^{\infty}\frac{d\omega}{2\pi}\big[S_{12}(\omega) + S_{21}(\omega)\big]F_c(\omega, t), \tag{8}$$

with the filter function $F_c(\omega, t) = [\cos\omega t - 1]/\omega$. We now further decompose the noise power spectral densities into classical and quantum components, arriving at:

$$\mathcal{J}(t) = \frac{2}{\hbar^2}\int_0^{\infty}\frac{d\omega}{2\pi}\big[S_{12}^Q(\omega) + S_{21}^Q(\omega)\big]F_c(\omega, t). \tag{9}$$

It is clear that the correlated classical noise spectral densities cancel out, suggesting that this coherent Ising interaction is solely determined by the correlated quantum noise. An alternative way to understand this is by referring to Eq. (7). The coherent coupling arises solely from the commutators of the noise operators E_i. This implies that the coupling $\mathcal{J}^z$ vanishes when only classical noise is present, as E_i can be treated as classical variables, and the commutators then vanish.

Dissipative evolution. The dissipative part is given by

$$\mathcal{L}_{ij}^z(t)\rho = \gamma_{ij}^z(t)\mathcal{D}_{ij}^z\rho, \;\text{ with }\; \gamma_{ij}^z(t) = \frac{1}{\hbar^2}\int_{-t}^t ds\, S_{ij}(s). \tag{10}$$

It is clear that $\gamma_{ii}^z(t)$ is the local dephasing noise determined by the auto noise correlator S_{ii}, whereas $\gamma_{12}^z(t)$ stands for the correlated dephasing governed by the cross noise correlator S_{12}. In terms of the noise spectral density, the pure-dephasing parameters can be written as

$$\gamma_{ij}^z(t) = \frac{2}{\hbar^2}\int_{-\infty}^{\infty}\frac{d\omega}{2\pi}S_{ij}(\omega)F_s(\omega, t), \tag{11}$$

with the filter function $F_s(\omega, t) = \sin \omega t/\omega$. When we further decompose the noise spectral densities into quantum and classical components, we arrive at:

$$\gamma_{ij}^z(t) = \frac{2}{\hbar^2} \int_0^\infty \frac{d\omega}{2\pi} F_s(\omega, t) \big\{ [S_{ij}^C(\omega) + S_{ji}^C(\omega)] + [S_{ij}^Q(\omega) - S_{ji}^Q(\omega)] \big\}. \tag{12}$$

This is the equation presented in the main text by using the fact that $S_{ij}^*(\omega) = S_{ji}(\omega)$. First, we observe that the local dephasing rate γ_{ii}^z is solely determined by the local classical noise $S_{ii}^C(\omega)$; the quantum component does not enter the local dephasing. In contrast, for the correlated dephasing process, the rate γ_{12}^z is governed by both $\mathrm{Re}\, S_{12}^C(\omega)$ and $\mathrm{Im}\, S_{12}^Q(\omega)$. As we discussed in the main text, $\mathrm{Im}\, S_{12}^Q$ vanishes unless the spectrum of the quasiparticle in the environment $\omega_{\mathbf{k}}$ is asymmetric. Otherwise, in the case of symmetric environment, both local and correlated dephasing processes are dictated by the classical noise, and the quantum noise only leads to the coherent Ising interaction between qubits.

Master equation for pure-transverse noise In this subsection, we derive the TCL master equations for the two qubit system in the presence of coherent drives at resonance. In this scenario, the coupling between the system and the environment is given by

$$H_{\mathrm{SE}}(t) = -\sum_i \hat{\sigma}_i^x E_i(t) = -\sum_i \big(e^{i\Omega t} \hat{\sigma}_i^+ + e^{-i\Omega t} \hat{\sigma}_i^- \big) E_i(t) \tag{13}$$

in the interaction representation with $\hat{\sigma}^\pm \equiv (\hat{\sigma}^x \pm i\hat{\sigma}^y)/2$. With this interaction, we can derive the master equation:

$$\begin{aligned} \frac{\mathrm{d}\rho(t)}{\mathrm{d}t} = -\frac{1}{\hbar^2} \sum_{ij} \int_0^t ds\, \big[& e^{i\Omega s} S_{ij}(s) (\hat{\sigma}_i^+ \hat{\sigma}_j^- \rho - \hat{\sigma}_j^- \rho \hat{\sigma}_i^+) \\ & + e^{-i\Omega s} S_{ij}(s) (\hat{\sigma}_i^- \hat{\sigma}_j^+ \rho - \hat{\sigma}_j^+ \rho \hat{\sigma}_i^-) + e^{-i\Omega s} S_{ij}(-s) \\ & (\rho \hat{\sigma}_i^+ \hat{\sigma}_j^- - \hat{\sigma}_j^- \rho \hat{\sigma}_i^+) + e^{i\Omega s} S_{ij}(-s) (\rho \hat{\sigma}_i^- \hat{\sigma}_j^+ - \hat{\sigma}_j^+ \rho \hat{\sigma}_i^-) \big]. \end{aligned} \tag{14}$$

We can then regroup the terms on the right hand side and rewrite the equation into the following compact form:

$$\dot{\rho}(t) = -i[H_{xy}(t), \rho(t)] + \sum_{i,j \in \{1,2\}} \mathcal{L}_{ij}(t) \rho(t). \tag{15}$$

We discuss below the time-dependent coherent interaction $H_{xy}(t)$ and the dissipative part, respectively.

Coherent interaction. The coherent coupling induced by the environment is given by $H_{xy}(t) = \mathcal{J}(t) \hat{\sigma}_1^+ \hat{\sigma}_2^- + \mathcal{J}^*(t) \hat{\sigma}_1^- \hat{\sigma}_2^+$ with time-dependent coupling strength being:

$$\begin{aligned} \mathcal{J}(t) &= \frac{1}{2i\hbar^2} \int_0^t ds\, \big[e^{i\Omega s} S_{12}(s) - e^{-i\Omega s} S_{12}(-s) \\ &\qquad + e^{-i\Omega s} S_{21}(s) - e^{i\Omega s} S_{21}(-s) \big] \\ &= \frac{1}{2\hbar^2} \int_0^t ds\, \big[\mathcal{G}_{12}^R(s) e^{i\Omega s} + \mathcal{G}_{21}^R(s) e^{-i\Omega s} \big]. \end{aligned} \tag{16}$$

We see that, again, the coupling can be expressed in terms of the retarded Green's function, suggesting that the coherent interaction is rooted in the correlated quantum noise, similar to the coherent Ising interaction we discussed before. This point becomes clear in the discussion below. Let us first express the coherent interaction in terms of the noise spectral density $S_{12}(\omega)$:

$$\mathcal{J}(t) = \frac{1}{\hbar^2} \int_{-\infty}^\infty \frac{d\omega}{2\pi} \big[S_{12}(\omega) F_c(\omega - \Omega, t) + S_{21}(\omega) F_c(\omega + \Omega, t) \big]. \tag{17}$$

We now further decompose the spectral density $S_{12}(\omega)$ into positive and negative part which can be expressed in terms of the quantum and classical noise. We finally arrive at

$$\mathcal{J}(t) = \frac{2}{\hbar^2} \int_0^\infty \frac{d\omega}{2\pi} \big[S_{12}^Q(\omega) F_c(\omega - \Omega, t) + S_{21}^Q(\omega) F_c(\omega + \Omega, t) \big]. \tag{18}$$

It is clear that this coherent coupling takes a similar form as the Ising coupling $\mathcal{J}^z(t)$, and at $\Omega \to 0$, we obtain $\mathcal{J}(t) = \mathcal{J}^z(t)$. However, we should point out one crucial difference between them: the Ising coupling is always real whereas the coherent coupling $\mathcal{J}(t)$ is complex in general consisting of a real part that describes the symmetry exchange between the two qubits and a imaginary part that represents the Dzyaloshinskii–Moriya (antisymmetric exchange) interaction.

Dissipative evolution. The dissipator in the master equation (15) is given by the following Lindbladians:

$$\begin{aligned}\mathcal{L}_{ij}\rho = \gamma_{ij}^{\downarrow}(t)\Big[\hat{\sigma}_j^-\rho\hat{\sigma}_i^+ - \frac{1}{2}\{\hat{\sigma}_i^+\hat{\sigma}_j^-,\rho\}\Big]\\ + \gamma_{ij}^{\uparrow}(t)\Big[\hat{\sigma}_j^+\rho\hat{\sigma}_i^- - \frac{1}{2}\{\hat{\sigma}_i^-\hat{\sigma}_j^+,\rho\}\Big],\end{aligned} \tag{19}$$

with rates being

$$\begin{aligned}\gamma_{ij}^{\downarrow}(t)=&\frac{1}{\hbar^2}\int_{-t}^{t} ds S_{ij}(\tau)e^{i\Omega\tau}=\frac{2}{\hbar^2}\int_{-\infty}^{\infty}\frac{d\omega}{2\pi}S_{ij}(\omega)F_s(\omega-\Omega,t),\\ \gamma_{ij}^{\uparrow}(t)=&\frac{1}{\hbar^2}\int_{-t}^{t} ds S_{ij}(\tau)e^{-i\Omega\tau}=\frac{2}{\hbar^2}\int_{-\infty}^{\infty}\frac{d\omega}{2\pi}S_{ij}(\omega)F_s(\omega+\Omega,t).\end{aligned} \tag{20}$$

Here terms with $i = j$ stand for the local emission and absorption processes governed by the local noise spectral density $S_{ii}(\omega)$, whereas the terms with $i \neq j$ represent the correlated emission and absorption processes rooted in the cross noise spectral density $S_{12}(\omega)$. In the long time dynamics $\Omega t \gg 1$, the filter function $F_s(\omega \pm \Omega, t)$ approaches a delta function $\pi\delta(\omega \pm \Omega)$. In this case, the decay and absorption rates become time-independent and we can approximate them with $\gamma_{ij}^{\downarrow} = S_{ij}(\Omega)/\hbar^2$ and $\gamma_{ij}^{\uparrow} = S_{ij}(-\Omega)/\hbar^2$. They are related by the Boltzmann factor, $\gamma_{ij}^{\downarrow} = e^{\beta\hbar\Omega}\gamma_{ji}^{\uparrow}$. This is the detailed balance condition. Specifically, at low temperatures, the decaying process dominates and we can approximate $\gamma_{ij}^{\uparrow} \approx 0$. To further illustrate the effects of the classical and quantum noise, we express the local and correlated decay rates in terms of the classical and quantum noise spectral densities, yielding:

$$\begin{aligned}\gamma_{ij}^{\downarrow}(t)=&\frac{2}{\hbar^2}\int_0^{\infty}\frac{d\omega}{2\pi}\Big[S_{ij}^C(\omega)F_s(\omega-\Omega,t)+S_{ji}^C(\omega)F_s(\omega+\Omega,t)\Big]\\ &+\frac{2}{\hbar^2}\int_0^{\infty}\frac{d\omega}{2\pi}\Big[S_{ij}^Q(\omega)F_s(\omega-\Omega,t)-S_{ji}^Q(\omega)F_s(\omega+\Omega,t)\Big].\end{aligned} \tag{21}$$

The local and correlated absorption rate $\gamma_{ij}^{\uparrow}(t)$ is given by the same expression above but with $\Omega \to -\Omega$. It is now clear that, in contrast to the pure dephasing dynamics, here the quantum noise leads to both local and correlated decoherence. We can similarly approximate the filter function with a delta function when $t \gg 1/\Omega$. We then arrive at $\gamma_{ij}^{\downarrow} = \big[S_{ij}^C(\Omega) + S_{ij}^Q(\Omega)\big]/\hbar^2$ and $\gamma_{ij}^{\uparrow} = \big[S_{ji}^C(\Omega) - S_{ji}^Q(\Omega)\big]/\hbar^2$. From these expressions, we conclude that the asymmetry between the decay and absorption processes is rooted in the quantum noise. In the absence of any quantum noise, decay and absorption occur with equal strength, corresponding to the infinite temperature limit.

At this point, it is beneficial to recapitulate the dimensions of some critical parameters and functions that are frequently used in the main text:

$$\begin{aligned}&[E_i] = \text{energy},\ \ [S_{ij}(t)] = \text{energy}^2,\ \ [F_{c,s}] = \text{time},\\ &[\gamma_{ij}] = \text{time}^{-1},\ \ [S_{ij}(\omega)] = \text{time}\cdot\text{energy}^2,\\ &[\mathcal{J}^z] = [\mathcal{J}] = \text{time}^{-1}.\end{aligned} \tag{22}$$

and the σ in the definition of the $1/f$ noise has the dimension of energy.

SUPPLEMENTARY NOTE 3: PURE DEPHASING DYNAMICS

In the section of Results, we study the pure dephasing dynamics when the two qubits are subjected to correlated classical and quantum $1/f$ noise. Here, we solve the corresponding TCL master equation analytically and derive some results used in the main text. For pure dephasing dynamics, it is convenient to work in the basis $|a\rangle \in \{|\uparrow\uparrow\rangle, |\uparrow\downarrow\rangle, |\downarrow\uparrow\rangle, |\downarrow\downarrow\rangle\}$ (eigenstates of $\sigma_1^z \otimes \sigma_2^z$). Let us denote the density matrix as $\rho(t) = \sum_{a,b} G_{ab}\,|a\rangle\langle b|$. Then all

density matrix elements are decoupled from each other with the following equation of motions:

$$\begin{aligned}
\dot{G}_{12} &= -2\big[\gamma^z(t) - i\,\mathrm{Im}\,\gamma^z_{12}(t)\big]G_{12} - 2i\mathcal{J}^z(t)G_{12}, \\
\dot{G}_{13} &= -2\big[\gamma^z(t) + i\,\mathrm{Im}\,\gamma^z_{12}(t)\big]G_{12} - 2i\mathcal{J}^z(t)G_{13}, \\
\dot{G}_{24} &= -2\big[\gamma^z(t) - i\,\mathrm{Im}\,\gamma^z_{12}(t)\big]G_{24} + 2i\mathcal{J}^z(t)G_{24}, \\
\dot{G}_{34} &= -2\big[\gamma^z(t) + i\,\mathrm{Im}\,\gamma^z_{12}(t)\big]G_{34} + 2i\mathcal{J}^z(t)G_{34},
\end{aligned} \tag{23}$$

which are dependent on the local classical noise and spatially correlated quantum noise. Here, we emphasize that the local dephasing parameter $\gamma^z(t)$ is determined solely by local classical noise, whereas both the imaginary part of correlated dephasing $\mathrm{Im}\,\gamma^z_{12}$ and the Ising coupling $\mathcal{J}^z$ are rooted in the imaginary and real parts of the correlated quantum noise spectral density S^Q_{12}, respectively. The remaining two off-diagonal elements are solely determined by local and spatially correlated classical noise:

$$\begin{aligned}
\dot{G}_{23} &= -4\big[\gamma^z(t) - \mathrm{Re}\,\gamma^z_{12}(t)\big]G_{23}, \\
\dot{G}_{14} &= -4\big[\gamma^z(t) + \mathrm{Re}\,\gamma^z_{12}(t)\big]G_{14},
\end{aligned} \tag{24}$$

where we recall that $\mathrm{Re}\,\gamma^z_{12}$ is dictated by the correlated classical noise only. Since all these equations are decoupled, one can easily solve them. Here we restrict to the scenario that we discuss in the main text: namely the correlated noise is comparable with the local noise $S^C_{12}(\omega) \approx e^{i\theta} S^C_{ii}(\omega)$ and we are in the quantum regime such that $S^Q \approx S^C$. Here, we assume the phase of the correlated noise spectral function is constant θ for simplicity. We then have $\gamma^z_{12} = e^{i\theta}\gamma^z$.

When the local classical noise is $1/f$, i.e. $S^C_{ii}(\omega) = 2\pi\sigma^2/|\omega|$, we can evaluate the Ising coupling $\mathcal{J}^z$ defined by Eq. (9):

$$\mathcal{J}^z(t) = \frac{4}{\hbar^2}\int_0^\infty \frac{d\omega}{2\pi}\cos\theta\frac{2\pi\sigma^2}{\omega}F_c(\omega,t) = -\frac{2\pi\sigma^2\cos\theta}{\hbar^2}t, \tag{25}$$

and the pure-dephasing parameter γ^z defined by Eq. (12):

$$\gamma^z(t) = \frac{4}{\hbar^2}\int_{\omega_l}^\infty \frac{d\omega}{2\pi}F_s(\omega,t)\frac{2\pi\sigma^2}{\omega} = \frac{4\sigma^2 t}{\hbar^2}[1-\mathrm{Ci}(\omega_l t)]. \tag{26}$$

Here, we have introduced a low frequency cutoff ω_l which is set by the experimental measurement time and the cosine integral function defined by:

$$\mathrm{Ci}(x) = -\int_x^\infty dt\frac{\cos t}{t} = \gamma + \ln x + \sum_{k=1}^{\infty}\frac{(-x^2)^k}{2k(2k)!}, \tag{27}$$

where γ is Euler's constant. Since the dynamics that we are interested in occurs within a time much shorter compared to the measurement time, i.e. $\omega_l t \ll 1$, we can approximate $\mathrm{Ci}(x) = \gamma + \ln x$ and we thus have $\gamma^z(t) \approx 4\sigma^2 t[1-\gamma-\ln(\omega_l t)]/\hbar^2$. When we solve the master equation, it is convenient to introduce two time-dependent functions,

$$\begin{aligned}
\Gamma^z(t) &\equiv \int_0^t ds\,\gamma^z(s) \approx \frac{\sigma^2 t^2}{\hbar^2}\big[3 - 2\gamma - 2\ln(\omega_l t)\big], \\
V(t) &\equiv \int_0^t ds\mathcal{J}^z(s) = -\frac{\pi\sigma^2 t^2\cos\theta}{\hbar^2}.
\end{aligned} \tag{28}$$

All elements can be expressed in terms of these two functions and are given by the following expressions:

$$\begin{aligned}
G_{12}(t) &= G_{12}(0)\exp[-2(1-i\sin\theta)\Gamma^z(t) - 2iV(t)], \\
G_{13}(t) &= G_{13}(0)\exp[-2(1+i\sin\theta)\Gamma^z(t) - 2iV(t)], \\
G_{24}(t) &= G_{24}(0)\exp[-2(1-i\sin\theta)\Gamma^z(t) + 2iV(t)], \\
G_{34}(t) &= G_{34}(0)\exp[-2(1+i\sin\theta)\Gamma^z(t) + 2iV(t)], \\
G_{23}(t) &= G_{23}(0)\exp[-4(1-\cos\theta)\Gamma^z(t)], \\
G_{14}(t) &= G_{14}(0)\exp[-4(1+\cos\theta)\Gamma^z(t)].
\end{aligned} \tag{29}$$

With the explicit expression of the reduced density matrix $\rho(t)$, one can evaluate the entanglement of the two-qubit system as a function of time with arbitrary initial state.

SUPPLEMENTARY NOTE 4: MARKOVIAN LIMIT

In Results of the main text, we discuss the dynamics of two qubits subjected to coherent drives with Markovian noise. In this section, we provide detailed derivations of some results used in the main text. We first introduce the concurrence as a measure of the entanglement of the two qubits in the symmetrized and antisymmetrized basis. We then present an analytical solution for the master equation in the absence of the DM interaction. Next, we discuss the two qubit dynamics in the absence of the symmetric exchange interaction. We then present a study of the two qubit system when both the symmetric exchange and DM interactions are present. Finally, we investigate the long-term dynamics of the two qubit system at finite temperatures.

The dynamics of the two-qubit system is governed by the master equation (15). In the case of Markovian noise, one can extend the time to infinity in the expression of the coherent coupling $\mathcal{J}$ given by Eq. (16) and decay and absorption rates $\gamma_{ij}^{\downarrow}(t), \gamma_{ij}^{\uparrow}(t)$ given by Eq. (20). We then have the following expressions:

$$\mathcal{J} = \frac{\mathcal{G}_{12}^{R}(\Omega) + \mathcal{G}_{21}^{R}(-\Omega)}{2\hbar^2}, \gamma_{ij}^{\downarrow} = \frac{S_{ij}(\Omega)}{\hbar^2}, \gamma_{ij}^{\downarrow} = \frac{S_{ij}(-\Omega)}{\hbar^2}. \tag{30}$$

One important relation is $\gamma_{ij}^{\downarrow} = e^{\beta\hbar\Omega}\gamma_{ji}^{\uparrow}$, which allows us to gauge out the phase of $\gamma_{ij}^{\downarrow}$ (and also $\gamma_{ij}^{\uparrow}$) and absorb the phase into the coherent coupling $\mathcal{J}$. Therefore, we will assume that $\gamma_{ij}^{\downarrow}$ and $\gamma_{ij}^{\uparrow}$ are positive-valued while $\mathcal{J}$ is complex in general.

It is convenient to work in the symmetrized and antisymmetrized basis: $|a\rangle \in \{|\uparrow\uparrow\rangle, |T\rangle, |S\rangle, |\downarrow\downarrow\rangle\}$, where $|T\rangle$ and $|S\rangle$ are the standard triplet and singlet states. In this basis, let us denote the density matrix as

$$\begin{aligned} \rho =& G_t |T\rangle\langle T| + G_s |S\rangle\langle S| + G_{11} |\uparrow\uparrow\rangle\langle\uparrow\uparrow| + G_{44} |\downarrow\downarrow\rangle\langle\downarrow\downarrow| \\ &+ G_{ts} |T\rangle\langle S| + G_{st} |S\rangle\langle T| + \Delta\rho, \end{aligned} \tag{31}$$

where $\Delta\rho$ stands for other off-diagonal elements. As we show below the dynamics of the density matrix elements that we introduce in the above expression are closed, we will explore the dynamics within this subspace. From the master equation (15), we deduce the equations of motion for the diagonal elements:

$$\begin{aligned} \dot{G}_{11} &= -2\gamma^{\downarrow}G_{11} + (\gamma^{\uparrow} + \gamma_{12}^{\uparrow})G_t + (\gamma^{\uparrow} - \gamma_{12}^{\uparrow})G_s, \\ \dot{G}_{44} &= (\gamma^{\downarrow} + \gamma_{12}^{\downarrow})G_t + (\gamma^{\downarrow} - \gamma_{12}^{\downarrow})G_s - 2\gamma^{\uparrow}G_{44}, \\ \dot{G}_t &= -2\mathcal{D}x + (\gamma^{\downarrow} + \gamma_{12}^{\downarrow})G_{11} - (\gamma^{\downarrow} + \gamma_{12}^{\downarrow})G_t \\ &\qquad + (\gamma^{\uparrow} + \gamma_{12}^{\uparrow})G_{44} - (\gamma^{\uparrow} + \gamma_{12}^{\uparrow})G_t, \\ \dot{G}_s &= 2\mathcal{D}x + (\gamma^{\downarrow} - \gamma_{12}^{\downarrow})G_{11} - (\gamma^{\downarrow} - \gamma_{12}^{\downarrow})G_s \\ &\qquad + (\gamma^{\uparrow} - \gamma_{12}^{\uparrow})G_{44} - (\gamma^{\uparrow} - \gamma_{12}^{\uparrow})G_s. \end{aligned} \tag{32}$$

Here are some remarks regarding these equations. Firstly, we denote the local decay (absorption) rates as $\gamma^{\downarrow,\uparrow} \equiv \gamma_{ii}^{\downarrow,\uparrow}$, which must be larger compared to their nonlocal counterparts as guaranteed by the complete positivity of the dynamics. Secondly, we note that the change rate of the summation of the diagonal elements vanishes, as expected, suggesting that the evolution is trace-preserving. Thirdly, since all the basis states are eigenstates of the term $\propto \mathcal{J}_s$ of the coherent Hamiltonian, this term does not contribute to the dynamics of the diagonal elements and only results in the oscillation of off-diagonal elements G_{ts}. Lastly, since the term proportional to $\mathcal{D}$ breaks the parity, it relates the dynamics G_t to G_s.

Let us introduce $G_{ts} \equiv x + iy$ for convenience. We can also write down the equation for the off-diagonal term:

$$\begin{aligned} \dot{x} &= -(\gamma^{\downarrow} + \gamma^{\uparrow})x + 2\mathcal{J}_s y + \mathcal{D}(G_t - G_s), \\ \dot{y} &= -(\gamma^{\downarrow} + \gamma^{\uparrow})y - 2\mathcal{J}_s x. \end{aligned} \tag{33}$$

As anticipated, these equations are closed. Our discussion below will focus on this closed subspace.

Concurrence in symmetrized and antisymmetrized basis. For a pure bipartite state $\rho_{\mathrm{AB}} = |\psi_{\mathrm{AB}}\rangle\langle\psi_{\mathrm{AB}}|$, we usually adopt the von Neumann entropy as the entanglement measure: $S(|\psi_{\mathrm{AB}}\rangle) \equiv -\operatorname{tr}\rho_A \ln\rho_A = -\operatorname{tr}\rho_B \ln\rho_B$. For a general mixed state ρ_{AB}, this von-Neumann entropy is no longer a good measure since the classical mixture in ρ_{AB} will have a nonzero contribution. We will adopt entanglement of formation as our entanglement measure.

The entanglement of formation is defined as

$$E_F(\rho_{\rm AB}) \equiv \min \sum_i p_i\, S(\left|\psi^i_{\rm AB}\right\rangle), \tag{34}$$

where the minimum is taken over all possible decompositions of $\rho_{\rm AB} = \sum_i p_i \left|\psi^i_{\rm AB}\right\rangle \left\langle\psi^i_{\rm AB}\right|$ and $S(\left|\psi^i_{\rm AB}\right\rangle)$ is the von Neumann entropy of the pure state $\left|\psi^i_{\rm AB}\right\rangle$. Physically, $E_F(\rho_{\rm AB})$ is the minimum amount of pure state entanglement needed to create the mixed state. This is extremely difficult to evaluate in general since we need to try all the decompositions. Quite remarkably an explicit expression of $E_F(\rho_{\rm AB})$ is given when both A and B are two-state systems (qubits). This exact formula is based on the often used two-qubit concurrence, which is defined as

$$\mathcal{C}(\rho) = \max\{0, \lambda_1 - \lambda_2 - \lambda_3 - \lambda_4\}, \tag{35}$$

where λ_i's are, in decreasing order, the square roots of the eigenvalues of the matrix $\rho(\sigma_y \otimes \sigma_y)\rho^*(\sigma_y \otimes \sigma_y)$, where ρ^* is the complex conjugate of ρ. The entanglement of formation is then given by [1]

$$E_F(\rho) = h\Big(\frac{1+\sqrt{1-\mathcal{C}^2}}{2}\Big), \tag{36}$$

with $h(x) = -x\log_2 x - (1-x)\log_2(1-x)$. $E_F(\rho)$ is monotonically increasing and ranges from 0 to 1 as $\mathcal{C}(\rho)$ goes from 0 to 1, so that one can take the concurrence as a measure of entanglement in its own right. When we write the density matrix as:

$$\rho = \sum_{i,j\in\{\uparrow,\downarrow\}} G_{ij} \left|ij\right\rangle \left\langle ij\right| + G_{23} \left|\uparrow\downarrow\right\rangle \left\langle\downarrow\uparrow\right| + G_{32} \left|\downarrow\uparrow\right\rangle \left\langle\uparrow\downarrow\right|, \tag{37}$$

and other off-diagonal elements vanish. The concurrence can be shown to be

$$\mathcal{C}[\rho] = 2\max\big\{0, |G_{23}| - \sqrt{G_{11}G_{44}}\big\}. \tag{38}$$

On the other hand, we know the subspace spanned by $\{\left|\uparrow\downarrow\right\rangle, \left|\downarrow\uparrow\right\rangle\}$ is linked to the subspace spanned by $\{\left|T\right\rangle, \left|S\right\rangle\}$ through the following relation:

$$\hat{\rho} = U^\dagger \rho U, \ \text{ with } \ U = \frac{1}{\sqrt{2}}\begin{bmatrix} 1 & 1 \\ 1 & -1 \end{bmatrix}, \tag{39}$$

where we note that U is both hermtian and unitary. Then we can easily show that:

$$G_{23} = \frac{G_t - G_s}{2} - i\,\mathrm{Im}\,G_{ts}. \tag{40}$$

Therefore, in the basis that we used in the main text, the concurrence is given by:

$$\mathcal{C}[\rho] = \max\big\{0, |G_t - G_s - 2i\,\mathrm{Im}\,G_{ts}| - 2\sqrt{G_{11}G_{44}}\big\}. \tag{41}$$

At zero temperature, the probability of staying in the state $\left|\uparrow\uparrow\right\rangle$ vanishes $G_{11} = 0$ if the initial state has zero probability in the $\left|\uparrow\uparrow\right\rangle$ state. Consequently, the concurrence is further reduced to the following simple expression:

$$\mathcal{C}[\rho] = |G_s - G_t + 2i\,\mathrm{Im}\,G_{ts}|. \tag{42}$$

Before we close this subsection, we highlight a quick and convenient method to evaluate the lower bound of entanglement of formation for a generic mixed state ρ [2, 3]. It is known that, for the Werner states [4]

$$\rho_F = F\left|S\right\rangle\left\langle S\right| + \frac{1-F}{3}\Big(\left|T\right\rangle\left\langle T\right| + \sum_{i=\pm}\left|\Phi_i\right\rangle\left\langle\Phi_i\right|\Big), \tag{43}$$

the entanglement of formation is determined by the singlet fidelity $F = \left\langle S\right|\rho_F\left|S\right\rangle$. Here $\left|S\right\rangle, \left|T\right\rangle$ are singlet and triplet states, and $\left|\Phi_\pm\right\rangle \equiv (\left|\uparrow\uparrow\right\rangle \pm \left|\downarrow\downarrow\right\rangle)/\sqrt{2}$ are the other two Bell states. The entanglement of formation of ρ_F is given by the following function [2, 3]:

$$H(F) = \begin{cases} h[1/2 + \sqrt{F(1-F)}], & \text{for } 1/2 < F \le 1; \\ 0, & \text{for } 0 \le F \le 1/2. \end{cases} \tag{44}$$

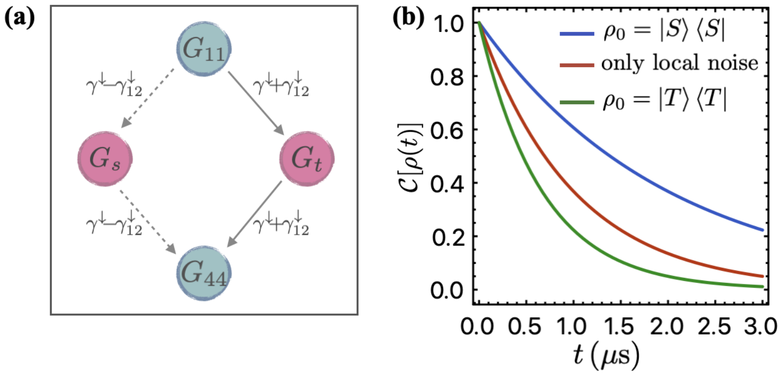

Supplementary Figure 1. (a) The decay of the state $|\uparrow\uparrow\rangle$ to $|\downarrow\downarrow\rangle$ can occur through two pathways. The first pathway is via the triplet state $|T\rangle$, with a decay rate of $\gamma^{\downarrow}+\gamma_{12}^{\downarrow}$. The second pathway is via the singlet state $|S\rangle$, with a decay rate of $\gamma^{\downarrow}-\gamma_{12}^{\downarrow}$. (b) When the DM interaction $\mathcal{D}$ is absent (i.e., $\mathcal{D}=0$), the entanglement between two qubits decays at a faster rate (the green curve) if the initial state is a triplet state $|T\rangle$, or decays at a lower rate (the blue curve) if the initial state is a singlet state $|S\rangle$, compared to the case when there is only local noise (the red curve, two initial states decay at the same rate). We assume a local decay rate of $\gamma^{\downarrow}=1$ GHz, and a correlated decay rate of $\gamma_{12}^{\downarrow}=0.5$ GHz.

Here, $h(x) \equiv -x\log_2 x - (1-x)\log_2(1-x)$. While the entanglement of formation of a generic mixed state ρ is not completely determined by the singlet fidelity F (we need to evaluate the relatively complicated quantity concurrence as we discussed above), the entangelment of formation of the corresponding Werner state [with $F=\langle S|\rho|S\rangle$] provides a lower bound on $E_F(\rho)$ [2, 3]:

$$H(F) \leq E_F(\rho). \tag{45}$$

In fact, this lower bound can be improved by setting $F = \max\langle e|\rho|e\rangle$, where the maximum is over all completely entangled states $|e\rangle$ [2, 3]. One can interpret F as the "fully entangled fraction" of state ρ. Therefore, if $F>1/2$ for a given state ρ, it is entangled. However, it is important to note that obtaining $F\leq 1/2$ does not conclusively prove the absence of entanglement, as $H(F)$ only provides a lower bound. One example is

$$\rho_0 = \frac{1}{2}|\uparrow\uparrow\rangle\langle\uparrow\uparrow| + \frac{1}{2}|S\rangle\langle S|. \tag{46}$$

We find $F=\langle S|\rho_0|S\rangle = 1/2$, but it is important to note that this state is entangled [with nonzero concurrence $\mathcal{C}(\rho_0)=1/2$], as it cannot be constructed from unentangled pure states.

Symmetric exchange interaction. We now restrict our discussion to the quantum regime where the absorption rates vanish $\gamma^{\uparrow}=\gamma_{12}^{\uparrow}=0$. In this scenario, the equations of motion for the density matrix elements are reduced to:

$$\begin{aligned} &\dot{G}_{11} = -2\gamma^{\downarrow}G_{11},\ \ \dot{G}_{44} = (\gamma^{\downarrow}+\gamma_{12}^{\downarrow})G_t + (\gamma^{\downarrow}-\gamma_{12}^{\downarrow})G_s,\\ &\dot{G}_t = -2\mathcal{D}x + (\gamma^{\downarrow}+\gamma_{12}^{\downarrow})G_{11} - (\gamma^{\downarrow}+\gamma_{12}^{\downarrow})G_t,\\ &\dot{G}_s = 2\mathcal{D}x + (\gamma^{\downarrow}-\gamma_{12}^{\downarrow})G_{11} - (\gamma^{\downarrow}-\gamma_{12}^{\downarrow})G_s\\ &\dot{x} = -\gamma^{\downarrow}x + 2\mathcal{J}_s y + \mathcal{D}(G_t - G_s),\ \ \dot{y} = -\gamma^{\downarrow}y - 2\mathcal{J}_s x. \end{aligned} \tag{47}$$

Notably, the state $|\uparrow\uparrow\rangle$ can decay to $|\downarrow\downarrow\rangle$ in two ways. The first way is through the triplet state, where we have the so-called superradiance with the decay rate $\gamma^{\downarrow}+\gamma_{12}^{\downarrow}$. The second way is through the singlet state, where we have the so-called subradiance with the decay rate $\gamma^{\downarrow}-\gamma_{12}^{\downarrow}$. This is sketched in Supplementary Figure 1 (a). This also suggests that the singlet state and the triplet state decay at different rates in the presence of correlated noise, which is illustrated in Supplementary Figure 1 (b).

When we turn off the DM interaction, $\mathcal{D}=0$, and assume the initial condition $\rho_0 = |\uparrow\downarrow\rangle\langle\uparrow\downarrow|$ (namely, $G_t = G_s = G_{ts} = 1/2$), we first easily see that $G_{11}=0$ for all times. Then the equations for G_t and G_s are decoupled from other elements (due to the absence of the parity-breaking interaction $\mathcal{D}$):

$$\dot{G}_t = -(\gamma^{\downarrow}+\gamma_{12}^{\downarrow})G_t,\ \ \dot{G}_s = -(\gamma^{\downarrow}-\gamma_{12}^{\downarrow})G_s, \tag{48}$$

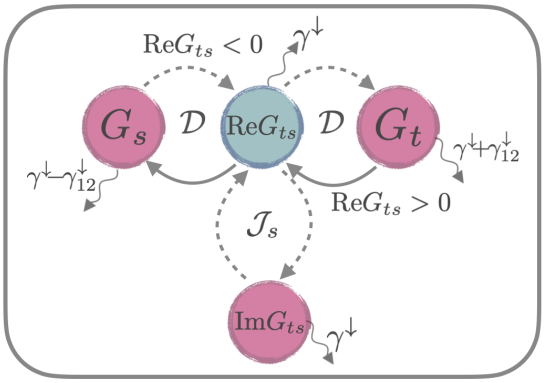

Supplementary Figure 2. Coupled dynamics of the populations in singlet state G_s and triplet state G_t governed by Eq. (47). The DM interaction, which breaks the parity symmetry and is assumed to be positive-valued $\mathcal{D} > 0$ for concreteness, couples the two states to each other. The flow of population from the triplet state to singlet state occurs when $\mathrm{Re}\, G_{ts}$ is positive and in the opposite direction when $\mathrm{Re}\, G_{ts}$ is negative. The dynamics of $\mathrm{Re}\, G_{ts}$ is determined by the relative populations of the singlet and triplet states, and is also coupled to $\mathrm{Im}\, G_{ts}$ via the symmetric exchange coupling $\mathcal{J}_s$.

which yield $G_t(t) = \exp[-(\gamma^{\downarrow} + \gamma^{\downarrow}_{12})t]/2$ and $G_s(t) = \exp[-(\gamma^{\downarrow} - \gamma^{\downarrow}_{12})t]/2$. By using the normalization $\mathrm{tr}\,\rho = 1$, we conclude that:

$$G_{44}(t) = 1 - e^{-\gamma^{\downarrow} t} \cosh\left(\gamma^{\downarrow}_{12} t\right). \tag{49}$$

For the off-diagonal element G_{ts}, we note that its real and imaginary parts x and y are coupled to each other. To this end, it is helpful to introduce $X(t) = x(t)e^{\gamma^{\downarrow} t}$ and $Y(t) = y(t)e^{\gamma^{\downarrow} t}$. The coupled equations then can be recast into the following compact form:

$$i\frac{\mathrm{d}}{\mathrm{d}t}\boldsymbol{\psi} = M\boldsymbol{\psi}, \tag{50}$$

with $\boldsymbol{\psi} \equiv (X, Y)^T$ and $M = -2\mathcal{J}_s\sigma_y$, which is solved by $\boldsymbol{\psi}(t) = \exp(-iMt)\boldsymbol{\psi}(0)$. By utilizing $\exp(-iMt) = \cos(2\mathcal{J}_s t) + \sigma_y \sin(2\mathcal{J}_s t)$, we arrive at $X(t) = \cos(2\mathcal{J}_s t)/2$ and $Y(t) = -\sin(2\mathcal{J}_s t)/2$, where we have used the initial condition $X(0) = 1/2$ and $Y(0) = 0$. Therefore, we have

$$G_{ts}(t) = \frac{\exp[-(\gamma^{\downarrow} + 2i\mathcal{J}_s)t]}{2}. \tag{51}$$

Thus, we have solved the master equation for the two-qubit system. In this case, we can write down the entanglement (concurrence) of the two qubits as a function of time:

$$\begin{aligned} \mathcal{C}[\rho(t)] &= |G_s - G_t + 2i\,\mathrm{Im}\, G_{ts}| \\ &= e^{-\gamma^{\downarrow} t}\sqrt{\sinh^2 \gamma^{\downarrow}_{12} t + \sin^2(2\mathcal{J}_s t)}. \end{aligned} \tag{52}$$

Dzyaloshinskii–Moriya interaction. Here, we turn off the symmetric exchange interaction $\mathcal{J}_s = 0$, and assume the initial state to be $|\uparrow\downarrow\rangle$. Similarly to the case above, we conclude that $G_{11}(t) = 0$. It is also clear that, since $\dot{y} = -\gamma^{\downarrow} y$, we have $y(t) = 0$ with the initial condition we assumed. Then all the equations are reduced to

$$\frac{\mathrm{d}}{\mathrm{d}t}\bar{\boldsymbol{\psi}} = \bar{M}\bar{\boldsymbol{\psi}}, \tag{53}$$

with $\bar{\boldsymbol{\psi}} = (G_t, G_s, x)^T$ and

$$M = \begin{bmatrix} -\Gamma_S & 0 & -2\mathcal{D} \\ 0 & -\Gamma_A & 2\mathcal{D} \\ \mathcal{D} & -\mathcal{D} & -\gamma^{\downarrow} \end{bmatrix}, \tag{54}$$

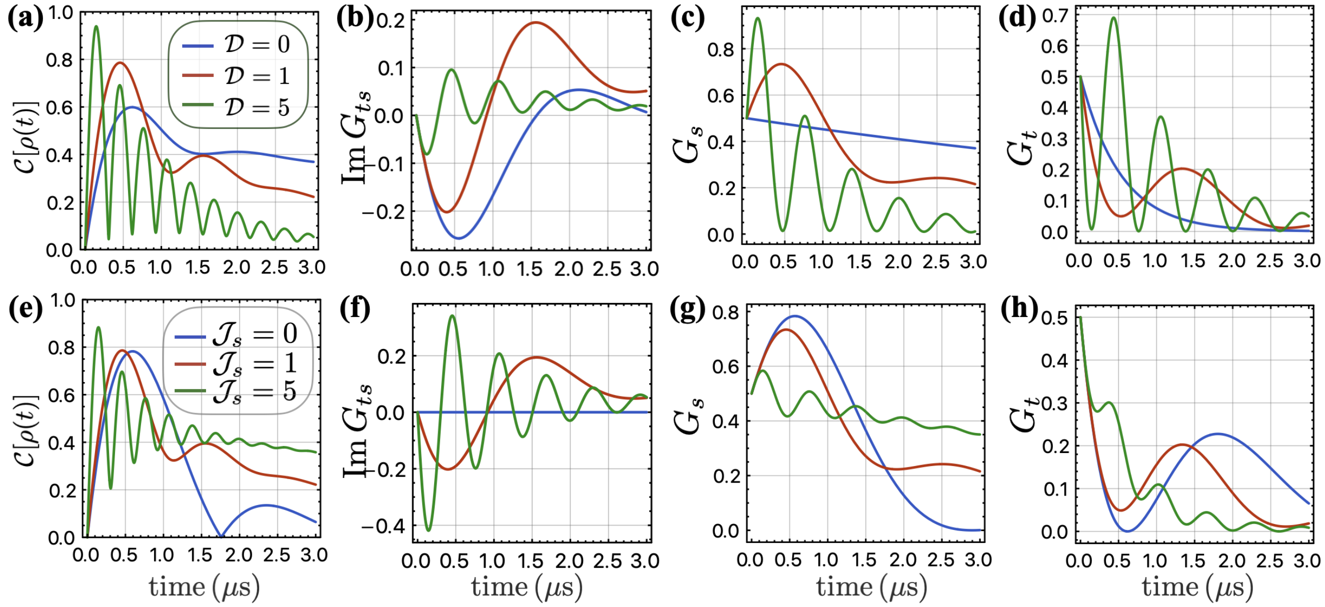

Supplementary Figure 3. Entanglement dynamics of resonantly driven qubits with initial state $|\uparrow\downarrow\rangle$ in the presence of both symmetric exchange and DM interaction. (a)-(d) Qubits dynamics with the fixed symmetric exchange coupling $\mathcal{J}_s = \gamma^{\downarrow}$ and varying DM interaction $\mathcal{D}$. (a) The entanglement measured by the concurrence $\mathcal{C}[\rho(t)]$ between two qubits is plotted as a function of time for varying strengths of $\mathcal{D}$. As $\mathcal{D}$ increases, the maximum entanglement also increases and the oscillation frequency of entanglement becomes faster. (b) The imaginary part of G_{ts} is shown as a function of time, which exhibits faster oscillations as $\mathcal{D}$ increases. Furthermore, the magnitude of $\mathrm{Im}\, G_{ts}$ is suppressed with an increase in $\mathcal{D}$. (c) The time-dependent function G_s is plotted, which displays a larger value at short times for higher values of $\mathcal{D}$, but has a smaller value overall with increasing $\mathcal{D}$. (d) The time-dependent function G_t is plotted, which exhibits both faster oscillations and larger magnitudes with increasing $\mathcal{D}$. (e)-(h) Qubits dynamics with the fixed DM interaction $\mathcal{D} = \gamma^{\downarrow}$, and varying symmetric exchange coupling $\mathcal{J}_s$.(e) The entanglement between two qubits is plotted as a function of time, and it exhibits faster oscillations for larger values of $\mathcal{J}_s$. Moreover, as $\mathcal{J}_s$ increases, the magnitude of entanglement increases, but the amount of increase is smaller than in (a). (f) The imaginary part of G_{ts} is shown as a function of time, which exhibits faster oscillations and larger amplitude as $\mathcal{J}_s$ is increased. (g) The time-dependent function G_s is plotted and displays faster oscillations for larger $\mathcal{J}_s$. Additionally, the magnitude of G_s is suppressed at shorter times but achieves a larger value at longer times as $\mathcal{J}_s$ is increased. (h) The time-dependent function G_t is plotted, which exhibits faster oscillations and a suppressed magnitude as $\mathcal{J}_s$ is increased. Parameters used in all figures: $\gamma^{\downarrow} = 1\,\mu\mathrm{s}^{-1}$ and $\gamma^{\downarrow}_{12} = 0.9\gamma^{\downarrow}$.

where $\Gamma_{S,A} = \gamma^{\downarrow} \pm \gamma^{\downarrow}_{12}$. To solve these three coupled differential equations, we convert them into a single third order differential equation. To this end, we first introduce $\bar{G}_t = G_t e^{\gamma^{\downarrow} t}, \bar{G}_s = G_s e^{\gamma^{\downarrow} t}, X = x e^{\gamma^{\downarrow} t}$. Then these equations are reduced to the following form:

$$\begin{aligned} \dot{\bar{G}}_t &= -\gamma^{\downarrow}_{12}\bar{G}_t - 2\mathcal{D}X, \\ \dot{\bar{G}}_s &= \gamma^{\downarrow}_{12}\bar{G}_s + 2\mathcal{D}X, \\ \dot{X} &= \mathcal{D}(\bar{G}_t - \bar{G}_s). \end{aligned} \tag{55}$$

By taking one more time derivative for X, we obtain a second order differential equation for X but not closed:

$$\ddot{X} = -\mathcal{D}\gamma^{\downarrow}_{12}(\bar{G}_t + \bar{G}_s) - 4\mathcal{D}^2 X. \tag{56}$$

We note that, when taking one more derivative, we have a closed third order differential equation for X:

$$\dddot{X} + (4\mathcal{D}^2 - \gamma^{\downarrow 2}_{12})\dot{X} = 0, \tag{57}$$

which can be solved easily together with the initial conditions: $\dot{X}(0) = 0, \ddot{X}(0) = -\mathcal{D}\gamma^{\downarrow}_{12} - 2\mathcal{D}^2$. For example, when $4\mathcal{D}^2 > \gamma^{\downarrow 2}_{12}$, we have:

$$\dot{X}(t) = -\frac{\mathcal{D}\gamma^{\downarrow}_{12} + 2\mathcal{D}^2}{\omega_r}\sin\omega_r t, \tag{58}$$

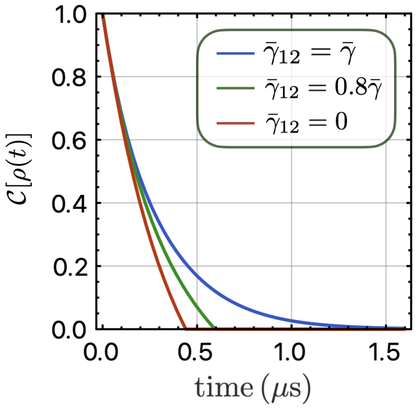

Supplementary Figure 4. Dynamics of entanglement between two qubits as a function of time in the presence of pure classical Markovian noise. The system is initialized in the Bell state $|\psi_0\rangle = (|\uparrow\downarrow\rangle + i\,|\downarrow\uparrow\rangle)/\sqrt{2}$. The curves correspond to the entanglement decay under different spatially correlated noise conditions. It is observed that the presence of correlated classical noise results in a slight modification of the decoherence rate, but does not introduce any new features. Parameters used in the plot: $\bar{\gamma} = 1\,\mu\text{s}^{-1}$.

with $\omega_r = \sqrt{4\mathcal{D}^2 - \gamma_{12}^{\downarrow 2}}$. It is then straightforward to obtain the analytic expressions for all the density matrix elements. Focusing on the dynamics of the entanglement between the qubits, one can easily write down its expression according to $\mathcal{C}[\rho(t)] = |G_s - G_t|$ once we have the explicit expression for the density matrix. Distinct two-qubit entanglement dynamics can be achieved with different values of the DM interaction $\mathcal{D}$. We also remark that the interplay between the DM interaction and the dissipative process can also lead to intriguing physics in classical dynamics.

On the other hand, it is helpful to write down the equation of motion for the entanglement directly and study how the quantum coherence in the system evolves. To this end, we introduce $\mathcal{C}_R \equiv G_s - G_t$ and we derive a differential equation for it. We note that it is linked to $\dot{X}$ via: $\mathcal{C}_R = -\dot{X}e^{-\gamma^{\downarrow}t}/\mathcal{D}$, from which we conclude that:

$$\ddot{\mathcal{C}}_R + 2\gamma^{\downarrow}\dot{\mathcal{C}}_R + (\gamma^{\downarrow 2} - \gamma_{12}^{\downarrow 2} + 4\mathcal{D}^2)\mathcal{C}_R = 0. \tag{59}$$

Symmetric and DM interactions. We illustrate the effects of the symmetric exchange and the DM interaction, respectively, by solving the master equation for the two qubits analytically. This allows us to extrapolate the scenario when both are present. In this case, all four important elements are coupled to each other, as shown in Supplementary Figure 2. The populations of the two states, G_t and G_s, are coupled to each other by the DM interaction (assumed to be positive for concreteness), which breaks the parity symmetry. Population flows from the triplet state to the singlet state when $\mathrm{Re}\,G_{ts}$ is positive, and in the opposite direction when it is negative. The dynamics of $\mathrm{Re}\,G_{ts}$ depend on the relative populations of the singlet and triplet states and are further coupled to $\mathrm{Im}\,G_{ts}$ through the symmetric exchange coupling $\mathcal{J}_s$.

In Supplementary Figure 3, we present the entanglement dynamics in two different scenarios. In the first case, we fix the symmetric exchange coupling $\mathcal{J}_s$ to be comparable to the local decay rate and gradually vary the strength of the DM interaction $\mathcal{D}$. Supplementary Figure 3 (a) demonstrates that as $\mathcal{D}$ increases, the maximal entanglement that can be achieved in the time evolution increases. This is what we expect since more probability flows to the singlet state before significant decoherence occurs, as illustrated in the plots for $G_s(t)$ and $G_t(t)$ in Supplementary Figure 3 (c) and (d), respectively. The green curves in (c) and (d) indicate that G_s reaches its first peak whereas G_t reaches its first valley after a certain time. For the red curves in (c) and (d), G_s reaches the peak at a later time and the peak is lower than the green curve. Additionally, the entanglement oscillation frequency increases as we increase $\mathcal{D}$, which is understandable as the coherent interaction generally leads to oscillation in the system (i.e., shuffle the information back and forth), as observed in Supplementary Figure 3 (b), (c), and (d), where all elements oscillate faster. Finally, we observe that the generated entanglement has a shorter lifetime, which is easy to understand since the DM interaction can bring the singlet state (leading to the residual entanglement) that decays slower to the triplet state that decays much faster. This is also evident in Supplementary Figure 3(c), where G_s decays much faster as the DM interaction increases.

In the second scenario, we assume that the DM interaction $\mathcal{D}$ is comparable to the local decay rate. Surprisingly, we observe that a increase in the symmetric exchange interaction does not lead to a significant increase in the maximum entanglement, as illustrated in Supplementary Figure 3 (e). This is because the symmetric exchange interaction

directly couples $\mathrm{Re}\, G_{ts}$ to $\mathrm{Im}\, G_{ts}$, whereas the maximum entanglement depends on the peak that G_s can reach, which is mainly determined by the value of $\mathcal{D}$. However, increasing $\mathcal{J}_s$ does lead to a faster oscillation in all elements, including the entanglement between the two qubits, as we can see in Supplementary Figure 3 (f), (g) and (h).

Pure classical noise. In Results of the main text, we mention that pure classical noise does not lead to any interesting dynamics. To illustrate this point, we first note that in the absence of any quantum noise, both symmetric exchange and DM interactions are absent, and the decay and absorption rates are equal, since their asymmetry is rooted in the quantum noise. We introduce the notations $\bar{\gamma} \equiv \gamma^{\uparrow} = \gamma^{\downarrow}$ for local decay and absorption, and $\bar{\gamma}_{12} \equiv \gamma_{12}^{\uparrow} = \gamma_{12}^{\downarrow}$ for the correlated decay and absorption. In this case, the coupled dynamics can be reduced to the following equations:

$$\begin{aligned}
\dot{G}_{11} &= -2\bar{\gamma}G_{11} + (\bar{\gamma}+\bar{\gamma}_{12})G_t + (\bar{\gamma}-\bar{\gamma}_{12})G_s, \\
\dot{G}_t &= (\bar{\gamma}+\bar{\gamma}_{12})G_{11} - 2(\bar{\gamma}+\bar{\gamma}_{12})G_t + (\bar{\gamma}+\bar{\gamma}_{12})G_{44}, \\
\dot{G}_s &= (\bar{\gamma}-\bar{\gamma}_{12})G_{11} - 2(\bar{\gamma}-\bar{\gamma}_{12})G_s + (\bar{\gamma}-\bar{\gamma}_{12})G_{44}, \\
\dot{G}_{44} &= (\bar{\gamma}+\bar{\gamma}_{12})G_t + (\bar{\gamma}-\bar{\gamma}_{12})G_s - 2\bar{\gamma}G_{44},
\end{aligned} \tag{60}$$

and the off-diagonal elements are decoupled from these diagonal elements: $\dot{x} = -2\bar{\gamma}x$ and $\dot{y} = -2\bar{\gamma}y$. Starting from these equations, it is straightforward to verify that the entanglement remains zero if the system is initialized to a product state. To demonstrate the impact of correlated classical noise on the decoherence process, we consider a specific initial state, the Bell state $|\psi_0\rangle = (|\uparrow\downarrow\rangle + i\,|\downarrow\uparrow\rangle)/\sqrt{2}$. Supplementary Figure 4 displays the entanglement decay for different strengths of correlated classical noise. Although the presence of classical noise modifies the decoherence process slightly, it does not introduce any new features. Therefore, in the main text, we concentrate on the quantum regime where both correlated classical and quantum noise coexist.

SUPPLEMENTARY NOTE 5: CORRELATED CLASSICAL AND QUANTUM $1/f$ NOISE

We first consider the case of purely classical $1/f$ noise and then consider the scenario where the quantum $1/f$ noise is comparable to the classical one. We assume the correlated noise is comparable to the local noise and is real valued $S_{12}(\omega) = S_{ii}$, as we assumed in the main text.

Correlated classical $1/f$ noise. In the presence of purely classical $1/f$ noise, the coherent coupling is absent. The decay and absorption rates take the following form from Eq. (21):

$$\begin{aligned}
\gamma_{ij}^{\downarrow}(t) &= \frac{2}{\hbar^2}\int_0^{\infty}\frac{d\omega}{2\pi}\Big[S_{ij}^{C}(\omega)F_s(\omega-\Omega,t) + S_{ji}^{C}(\omega)F_s(\omega+\Omega,t)\Big], \\
\gamma_{ij}^{\uparrow}(t) &= \frac{2}{\hbar^2}\int_0^{\infty}\frac{d\omega}{2\pi}\Big[S_{ji}^{C}(\omega)F_s(\omega-\Omega,t) + S_{ij}^{C}(\omega)F_s(\omega+\Omega,t)\Big].
\end{aligned} \tag{61}$$

With the assumption that $S_{ij}^{C}(\omega) = 2\pi\sigma^2/|\omega|$, we have the equal (local and correlated) absorption and decay rates [denoted as $\gamma(t) \equiv \gamma_{ij}^{\downarrow}(t) = \gamma_{ij}^{\uparrow}(t)$], which is given by

$$\begin{aligned}
\gamma(t) &= \frac{2}{\hbar^2}\int_{\omega_l}^{\infty}\frac{d\omega}{2\pi}\frac{2\pi\sigma^2}{\omega}\big[F_s(\omega-\Omega,t) + F_s(\omega+\Omega,t)\big] \\
&= \frac{4\sigma^2}{\hbar^2\Omega}\big[\mathrm{Si}(\Omega t) - \sin(\Omega t)\mathrm{Ci}(\omega_l t)\big].
\end{aligned} \tag{62}$$

One surprising feature is that the above decoherence rate can be temporarily negative. Here we have introduce the low frequency cutoff ω_l for the $1/f$ noise. For the purely classical $1/f$ noise, the system is governed by the same set of equations (60) with $\bar{\gamma} = \bar{\gamma}_{12} = \gamma(t)$. First, we can obtain the expression for $x(t)$ and $y(t)$ easily as they are decoupled from other elements:

$$x(t) = x(0)e^{-2\Gamma(t)}, \;\text{ and }\; y(t) = y(0)e^{-2\Gamma(t)}, \tag{63}$$

where we have introduced $\Gamma(t) = \int_0^t ds\,\gamma(s)$. We now are interested in how the entanglement decays with the decoherence rate $\gamma(t)$. To be specific, we assume the initial state is a Bell state $|\psi_0\rangle = (|\uparrow\downarrow\rangle + i\,|\downarrow\uparrow\rangle)/\sqrt{2}$, or equivalently, $G_t(0) = G_s(0) = y(0) = 1/2$ (other elements vanish). By using the symmetry between the absorption

and decay processes, we conclude that $G_{11}(t) = G_{44}(t)$. In the case of large correlated noise (comparable to local noise), $G_s(t) = 1/2$ remains to be a constant. From the fact $\operatorname{tr}\rho = 1$, we have the relation $G_t(t) = 1/2 - 2G_{11}$. We can deduce the equation for G_{11}:

$$\dot{G}_{11}(t) = -6\gamma(t)G_{11}(t) + \gamma(t), \tag{64}$$

which is solved to be

$$G_{11}(t) = \int_0^t d\tau \left\{\gamma(\tau)\exp\left[-6\int_\tau^t ds\,\gamma(s)\right]\right\}. \tag{65}$$

This can be simplified to $G_{11}(t) = [1 - \exp[-6\Gamma(t)]]/6$. One can similarly show that $G_t(t) = 1/6 + \exp[-6\Gamma(t)]/3$. Then one can evaluate the entanglement according to Eq. (41). When the initial state is a trivial product state, for example $|\uparrow\downarrow\rangle$ (namely, $G_t(0) = G_s(0) = y(0) = 1/2$), one can also show that the entanglement remains to be zero with the pure classical $1/f$ noise.

Correlated quantum $1/f$ noise. In the presence of correlated quantum noise $S^Q \approx S^C$, the coherent coupling $\mathcal{J}$ is finite (we assume the spectral density is real), which is evaluated to be:

$$\begin{aligned}\mathcal{J}(t) &= \frac{2}{\hbar^2}\int_0^\infty \frac{d\omega}{2\pi}\frac{2\pi\sigma^2}{\omega}\left[F_c(\omega-\Omega,t) + F_c(\omega+\Omega,t)\right] \\ &= -\frac{2\pi\sigma^2}{\hbar^2\Omega}\sin\Omega t,\end{aligned} \tag{66}$$

where we have taken the principle value of the integral. As we discussed in the main text, we approximate the absorption rate with zero. In this case, the two-qubit dynamics is governed by the same set of equations in Eq. (47) but with $\mathcal{J}_s = \mathcal{J}(t), \gamma^\downarrow = \gamma_{12}^\downarrow$ and $\mathcal{D} = 0$. We again consider two initial states: one is the Bell state $|\psi_0\rangle$ and the other one is the product state $|\uparrow\downarrow\rangle$. In both cases, we can show that $G_s(t) = 1/2$ and $G_t(t) = e^{-2\Gamma^\downarrow(t)}/2$ with $\Gamma^\downarrow(t) = \int_0^t ds\gamma^\downarrow(s)$. For the dynamics of $\operatorname{Re}G_{ts}$ and $\operatorname{Im}G_{ts}$, we introduce $X(t) \equiv xe^{\Gamma^\downarrow(t)}$ and $Y(t) \equiv ye^{\Gamma^\downarrow(t)}$. One can show that they are described by the following equations:

$$\frac{\mathrm{d}}{\mathrm{d}t}\begin{bmatrix}X(t)\\Y(t)\end{bmatrix} = \begin{bmatrix}0 & 2\mathcal{J}(t)\\ -2\mathcal{J}(t) & 0\end{bmatrix}\begin{bmatrix}X(t)\\Y(t)\end{bmatrix}, \tag{67}$$

from which we can obtain: $[X(t), Y(t)]^T = U(t)[X(0), Y(0)]^T$ with the rotation matrix:

$$U(t) = \begin{bmatrix}\cos\Phi(t) & -\sin\Phi(t)\\ \sin\Phi(t) & \cos\Phi(t)\end{bmatrix}, \tag{68}$$

where $\Phi(t) = \int_0^t ds\,\mathcal{J}(t)$. When the initial state is $|\uparrow\downarrow\rangle$, we have initial condition $X(0) = 1/2$ and $Y(0) = 0$ and obtain:

$$y(t) = \frac{\sin\Phi(t)}{2}\exp\left[-\Gamma^\downarrow(t)\right]. \tag{69}$$

When the initial state is the Bell state $|\psi_0\rangle$, we have $X(0) = 0$ and $Y(0) = 1/2$, which gives us

$$y(t) = \frac{\cos\Phi(t)}{2}\exp\left[-\Gamma^\downarrow(t)\right]. \tag{70}$$

So far, we have focused on the quantum regime, and it was shown in the main text that the final entanglement in this case is $1/2$. However, we also wish to examine the final entanglement as a function of temperature, or equivalently, the ratio between the quantum noise and classical noise, by invoking the following relation

$$\cosh\beta\hbar\Omega = \frac{[S^C(\Omega)]^2 + [S^Q(\Omega)]^2}{[S^C(\Omega)]^2 - [S^Q(\Omega)]^2}. \tag{71}$$

To this end, we set $\dot{\rho} = 0$ and $t \to \infty$ [then the dynamics is governed by Eq. (32) with all coefficients being constant in this limit]. We still assume the correlated noise is comparable to the local noise (otherwise, one can show the entanglement will eventually decay to zero). Then we can show that $G_s = 1/2$ and $G_{11} = \alpha G_t = \alpha^2 G_{44}$ with $2G_{44} = (1 + \alpha + \alpha^2)^{-1}$ and $\alpha = e^{-\beta\hbar\Omega}$ and other off-diagonal elements are zero.

* ji.zou@unibas.ch

Supplementary References

[1] W. K. Wootters, Entanglement of formation of an arbitrary state of two qubits, Phys. Rev. Lett. **80**, 2245 (1998).

[2] G. Burkard and D. Loss, Lower bound for electron spin entanglement from beam splitter current correlations, Physical review letters **91**, 087903 (2003).

[3] C. H. Bennett, D. P. DiVincenzo, J. A. Smolin, and W. K. Wootters, Mixed-state entanglement and quantum error correction, Physical Review A **54**, 3824 (1996).

[4] R. F. Werner, Quantum states with einstein-podolsky-rosen correlations admitting a hidden-variable model, Phys. Rev. A **40**, 4277 (1989).
